# Hundreds of genetic barcodes of the species-rich hydroid superfamily Plumularioidea (Cnidaria, Medusozoa) provide a guide toward more reliable taxonomy

**DOI:** 10.1038/s41598-018-35528-8

**Published:** 2018-12-20

**Authors:** Carlos J. Moura, Harilaos Lessios, Jorge Cortés, Martha S. Nizinski, John Reed, Ricardo S. Santos, Allen G. Collins

**Affiliations:** 1 MARE-IMAR-OKEANOS, Rua Prof. Dr Frederico Machado, 4, University of the Azores, Horta, 9901-862 Portugal; 2 Smithsonian Tropical Research Institute, Balboa, 0843-03092 Panamá USA; 3 National Systematics Laboratory, NOAA’s National Marine Fisheries Service, Smithsonian National Museum of Natural History, Washington, DC 20560 USA; 4 Centro de Investigación en Ciencias del Mar y Limnología (CIMAR), Universidad de Costa Rica, San Pedro, 11501-2060 San José, Costa Rica; 5 Harbor Branch Oceanographic Institute, Florida Atlantic University, Fort Pierce, Florida 34946 USA; 6 Present Address: CBMA – Centre of Molecular and Environmental Biology, Department of Biology, University of Minho, Campus de Gualtar, 4710-057 Braga, Portugal

## Abstract

Marine hydroids are important benthic components of shallow and deep waters worldwide, but their taxonomy is controversial because diagnostic morphological characters to categorize taxa are limited. Their genetic relationships are also little investigated. We tested taxonomic hypotheses within the highly speciose superfamily Plumularioidea by integrating a classical morphological approach with DNA barcoding of the 16S and COI mitochondrial markers for 659 and 196 specimens of Plumularioidea, respectively. Adding Genbank sequences, we inferred systematic relationships among 1,114 plumularioids, corresponding to 123 nominal species and 17 novel morphospecies in five families of Plumularioidea. We found considerable inconsistencies in the systematics of nominal families, genera and species. The families Kirchenpaueriidae and Plumulariidae were polyphyletic and the Halopterididae paraphyletic. Most genera of Plumularioidea are not monophyletic. Species diversity is considerably underestimated. Within our study, at least 10% of the morphologically-distinctive morphospecies are undescribed, and about 40% of the overall species richness is represented by cryptic species. Convergent evolution and morphological plasticity therefore blur systematic relationships. Additionally, cryptic taxa occur frequently in sympatry or parapatry, complicating correspondence with type material of described species. Sometimes conspecificity of different morphotypes was found. The taxonomy of hydroids requires continued comprehensive revision.

## Introduction

Hypothesizing common ancestry of each group of taxa in a hierarchic ordering, taxonomists seek diagnostic morphological (but also physiological and molecular) homologous characters for each cluster, and name the resulting assemblages. While it may seem straight forward to find diagnostic differences between the many forms of life, taxonomists frequently have difficulties identifying unambiguous and objective criteria to differentiate morphologically similar but distinct taxa. The criteria to delineate species or higher taxa are frequently subjective^1,2^. Therefore, the systematics of many taxa remain troublesome. Nevertheless, taxonomic effort has great value. Accurate and comprehensive systematics is critically needed for all research disciplines of biological sciences because species are the basic units of biodiversity. Erroneous species boundaries or biodiversity estimates may lead to incorrect answers to the questions scientists try to answer in various biological fields, ranging from conservation, ecology, bioprospecting to fisheries management^3^.

DNA sequencing is a powerful tool that provides access to numerous characters that may aid in differentiating between morphologically similar taxa. The cytochrome *c* oxidase subunit I (COI) has been recommended as a nearly universal standard DNA Barcode marker^4,5^. However, the mitochondrial small ribosomal subunit (16S) is the preferred DNA Barcode for the diverse groups of cnidarians in the Class Hydrozoa^6,7^. Not only is 16S much easier to amplify^6,8–10^, but no COI primers that universally target all hydrozoan clades have been designed to date. Additionally, 16S is much more informative of phylogenetic relationships, and thus is applicable for taxonomic levels ranging from populations to families (e.g.^6–25^, present study). Furthermore, the phylogenetic relationships derived from the 16S marker alone have been notably congruent with phylogenetic inferences based on multi-marker analyses^8–10,13,15,20,23–26^.

**Figure 1:**
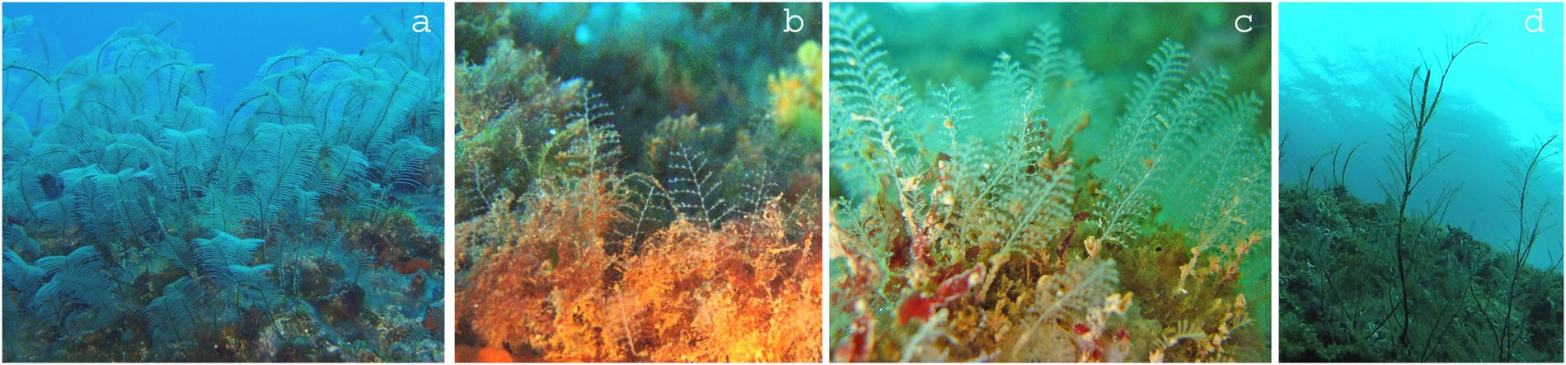
Examples of Plumularioidea hydroids: (**a**) *Aglaophenia* cf. *pluma* - Aglaopheniidae; (**b**) *Halopteris* sp. - Halopterididae; (**c**) *Kirchenpaueria pinnata* - Kirchenpaueriidae; (**d**) *Dentitheca dendritica* - Plumulariidae. Credits: Carlos J. Moura.

In this study, we sequenced fragments of both the 16S and COI mitochondrial genes from many members of the highly speciose superfamily Plumularioidea (Order Leptothecata, Class Hydrozoa; Fig. 1). According to the World Register of Marine Species (“WoRMS”) database^27^, the Plumularioidea comprise 583 nominal species, ordered into five nominal families: Aglaopheniidae (13 genera and 256 species), Schizotrichidae (1 genus and 23 species), Kirchenpaueriidae (6 genera and 46 species), Halopterididae (14 genera and 105 species) and Plumulariidae (9 genera and 153 species). The Plumularioidea are common components of benthic marine habitats worldwide, ranging from coastal to deep waters. The pelagic “medusa” phase is generally suppressed from the life cycle of plumularioids^28^. Only a few examples are known of species that release medusoids (rudimentary, short-lived and non-feeding, but reproductive medusae). Therefore, dispersal of the majority of the Plumularioidea should be fairly constrained by the release to the water column of gametes and short-lived planula larvae. However, dispersal may be enhanced by rafting and sporadic detachment from substrates of some species. Many Plumularioidea can develop relatively large sessile colonies that increase the structural complexity of habitats. Epibionts, including other hydroids, polychaetes, small crustaceans, sponges, bryozoans, protists and bacteria are frequently found associated with plumularioid colonies. Despite the general ubiquity and prominent colony sizes of many plumularioid species, the taxonomy of Plumularioidea has been highly disputed and unsettled. For example, WoRMS^27^ contains 395 unaccepted species names for this superfamily. Previous molecular analyses of some assemblages of Plumularioidea have revealed taxonomic inconsistencies, including polyphyletic or paraphyletic groupings, synonymies and cryptic lineages^6,9,16,18,19,23–25^. Taxonomy of hydrozoans is difficult because they are morphologically simple organisms with few diagnostic characters, high phenotypic plasticity, and many imprecisely described and/or synonymized taxa. As Plumularioidea are some of the better-known and studied hydrozoans, such systematic problems likely pertain to many of the other less-studied and morphologically simpler hydrozoans.

The present study contributes the most comprehensive phylogenetic reconstruction of the Plumularioidea ever attempted. Given that every species name represents a hypothesis, this study seeks to understand the validity of existing species names and richness represented by Plumularioidea. We test taxonomic relationships within the group through the integration of morphologic characters and molecular phylogenetic analyses of two mitochondrial genetic markers - 16S and COI. Using multiple available methods for molecule-based species delimitation, we sought to determine how accurate current taxonomy represents actual species diversity. Our results show that species diversity is much higher than current taxonomy would suggest. Our barcode data indicate that cryptic diversity is extremely common in supposedly low dispersing hydrozoans, reinforcing and magnifying findings from previous studies. We thus propose desirable taxonomic practices to clarify the systematics of the superfamily Plumularioidea, which in general should be applied to the phylum Cnidaria or other problematic taxonomic groups. We also investigate geographic and bathymetric distributions of some species.

## Methods

This study examines phylogenetic relationships between 1,114 colonies representing the five families of Plumularioidea collected worldwide from various depths. Twenty-one nominal genera were included in this study: one for the family Schizotrichidae, eight for Aglaopheniidae, three for Kirchenpaueriidae, five for Halopteriidae, and four for the family Plumulariidae. Taxa were identified morphologically into 123 nominal species and 17 unknown (likely new) morphospecies: 57 and 10 for Aglaopheniidae; six and none for Schizotrichidae; 11 and none for Kirchenpaueriidae sensu strictu (s.s.); 20 and four for Halopterididae s.s.; and 29 and three for Plumulariidae s.s. (cf. Supplementary Table S1).

### Taxon Sampling

**Figure 2:**
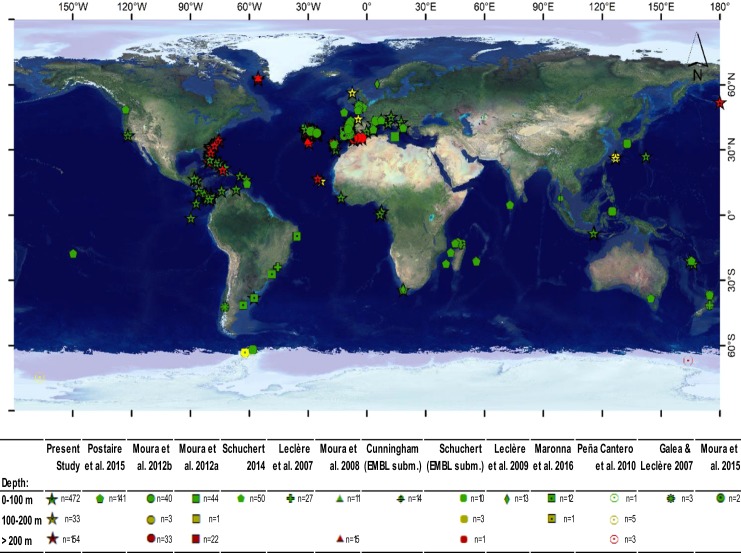
Geographical location, depth range and authorship of DNA sequences used in this study. The map was generated using ArcGIS 10.3.1 (http://www.esri.com/software/arcgis).

Samples were collected worldwide, but greater effort was invested in Central and North America, the Azores and a few locations off western Africa (Fig. 2). C.J.M. collected most of the shallow-water specimens from these regions by scuba diving or snorkeling, mainly between 2013 and 2014. He also accessed other samples collected through a great variety of methods by other researchers and stored in different institutions (see Supplementary Table S1 and Fig. 2). The specimens were preserved in 75–95% ethanol as soon as possible after sampling. In the laboratory, samples were identified to species according to morphology. Next, samples were genetically barcoded. After preliminary examination of molecular phylogenies, the morphology was re-examined. Samples were identified by C.J.M. based on taxonomic descriptions and according to the most up-to-date diagnoses and taxonomic decisions^27–35^. D. Calder reviewed some difficult taxonomic assignments. Vouchers were deposited in museums of different institutions according to the origin of specimens (see Supplementary Table S1). Photographs of vouchers of unusual species and of most putative species collected for this study were deposited in the repository www.morphbank.net (see Supplementary Table S1). The Smithsonian has engaged in a small digitization project that will associate photos of all specimens from this study with their respective USNM catalog numbers and make them accessible via their online catalog (https://collections.nmnh.si.edu/search/iz/.

### DNA extraction and sequencing

Molecular laboratory work was performed at the Laboratories of Analytical Biology in the National Museum of Natural History, Smithsonian Institution. Samples were digested overnight in buffer containing proteinase-k (1 mg/ml) before extraction. Total DNA was extracted using the AutoGenPrep phenol-chloroform automated extractor (AutoGen) from selected pieces of Plumularioidea colonies approximately 0.5–1 cm high. Primers of Cunningham and Buss^11^ were used to amplify ca. 600 base pairs of the mitochondrial 16 S rRNA. Sections of the cytochrome oxidase subunit I (COI) gene were amplified using primers LCO1490 and HCO2198^36^, and a new set of primers (designed by E. Kayal and C.J. Moura) “Cni_cox1_f1”: 5′- ACCNGAYATGGCNTTYCCNMG-3′, and “Cni_cox1_r1”: 5′-NATRTANACYTCNGGRTGNCC-3′.

A standard reaction mix (10 µL) contained 0.1 ul Taq (Biolase DNA polymerase (Bioline USA Inc., Taunton, MA), 0.3 ul of each primer, 0.5 ul dNTPs (Bioline), 0.6 ul magnesium chloride (Bioline), 2.5 ul BSA (New England BioLabs Inc., Ipswich, MA), and 1 ul of 10x Buffer (Bioline), 0.1 ul template DNA extraction, and 6.85 ul purified H_2_0. PCR cycling conditions for all pairs of primers were as follows: an initial denaturation at 95 °C for 5 min, followed by 40 cycles of amplification (denaturation at 95 °C for 45 s, annealing at 46°–50 °C for 45 s, and extension for 1 min at 72 °C), with final elongation step at 72 °C for 3 min. PCR products were checked by electrophoresis on 1.5% agarose gels, purified using ExoSAP-IT (Affymetrix, USB Products), Sanger sequenced in both directions using Big Dye (v. 3.1) and run on a 3730xl DNA sequencer (Applied Biosystems). Sequences were aligned, checked for sequencing errors and pruned to the length of the smallest fragment in Geneious v. 10.0.2^37^. GenBank accession numbers are listed in Supplementary Table S1. 16S sequences of another 455 Plumularioidea individuals^6,9,16,18,19,21,23,24,38–40^ and COI sequences of 55 additional *Plumularia* specimens^9^ were downloaded from GenBank (see Supplementary Table S1). Sequences of Sertulariidae individuals and of one Campanulariidae species were also downloaded from Genbank, to serve as outgroups for the alignments only with 16S sequences and alignments with COI and 16S sequences combined, respectively (see Supplementary Table S1).

### Phylogenetic analyses

Multiple sequence datasets, were incorporated in Geneious R10.0.2^37^, aligned with MAFFT^41^ (algorithm: Auto; scoring matrix: 200PAM/K = 2; gap open penalty = 1), and prepared for phylogenetic analyses. Datasets included: all 16S sequences, all COI and 16S sequences combined, 16S sequences for each family separately, and COI plus 16S sequences combined for each family separately. Subsequently, for the datasets with families separated, we used Gblocks server^42^ to obtain alignments with and without positions with gaps, and without “many contiguous nonconserved positions” (check the number of sequences and nucleotide positions used for each alignment in Supplementary Table S2).

Phylogenetic reconstructions included Maximum Likelihood (ML) and Bayesian Inference (BI) tree searches for all the alignments generated. The General Time Reversible model of nucleotide evolution with gamma and invariant parameters (GTR + G + I) was used for all analyses. ML analyses were conducted in PhyML^43^ (version 20120412), with 1000 bootstrap replications. Bayesian analyses were performed with MrBayes v.3.2.2^44^ and consisted of two runs of four chains each of 100 million generations with trees sampled every 1000 generations after a burn-in fraction of 0.25.

### Species delineation

We used two methods of species delineation, applied to the alignment containing all Plumularioidea sequences together: (i) the Automatic Barcode Gap Discovery (ABGD) method of Puillandre *et al*.^45^; (ii) the Poisson Tree Processes (PTP) method of Zhang *et al*.^46^. The ABGD method, performed via the website http://wwwabi.snv.jussieu.fr/public/abgd/, seeks to detect the ‘barcode gap’ based on the distribution of genetic pairwise distances. We selected the most conservative result from the ABGD analysis, i.e., the result that separated obvious morphological species and did not over split nominal species. The PTP method, which incorporates genetic distances with phylogenetic relationships to delimit species, was run via the PTP website http://species.h-its.org/, with ML and Bayesian approaches, using as input the ML tree without Gblocks manipulations and without outgroups. These analyses were run for 5 × 10^5^ MCMC generations, with a thinning value of 100 and a burn-in of 25%.

The putative species proposed were generally defined through the consensus of the most supported outputs of the different species delimitation methods. However, the morphological resemblance or divergence noticed between vouchers of each clade was also considered. We further provided degrees of uncertainty to the species delimitation results taking into account knowledge of the morphological diversity of lineages, and the probabilities output from the PTP analyses (Supplementary Table S1 and Fig. S1).

## Results and Discussion

**Figure 3:**
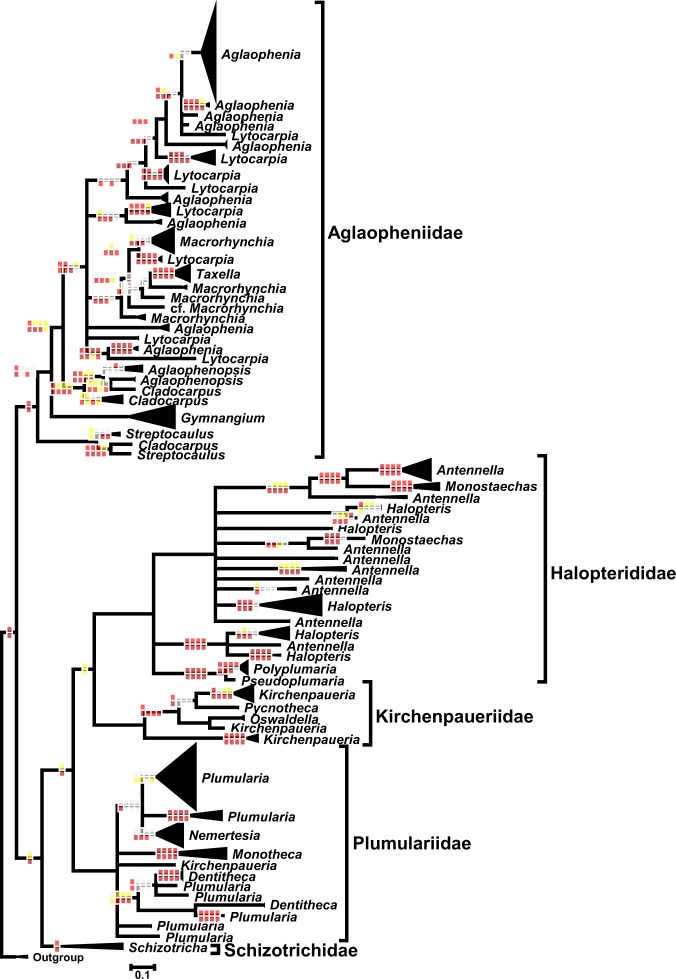
Molecular phylogeny of Plumularioidea based on 16S sequence data, without Gblocks manipulations, obtained with MrBayes. Nodes collapsed presented posterior probabilities below 70% for the phylogenetic analysis represented graphically. Rectangles on nodes show nodal support (for ML analyses: yellow – 50–74%; red – 75–94%; dark-red – 95–100%; for Bayesian analyses: yellow – 75–89%; red – 90–97%; dark-red – 98–100%) for the different phylogenetic analyses: first horizontal row – ML; second horizontal row – Bayesian; first vertical row – alignment with all families together with gaps; second vertical row – alignment without manipulations per family; third vertical row – alignment with some gaps removed, per family; fourth vertical row – alignment without gaps, per family. When nodal support is not indicated, all phylogenetic analyses achieved 95–100% of nodal support. See detailed composition of branches in Supplementary Fig. S1.

We evaluated phylogenetic relationships among 1,114 16S sequences of distinct Plumularioidea colonies, and obtained 676 unique haplotypes. Figure 3 and Supplementary Fig. S1 present a phylogenetic reconstruction of Plumularioidea haplotypes as inferred from 16S sequence data.

**Figure 4:**
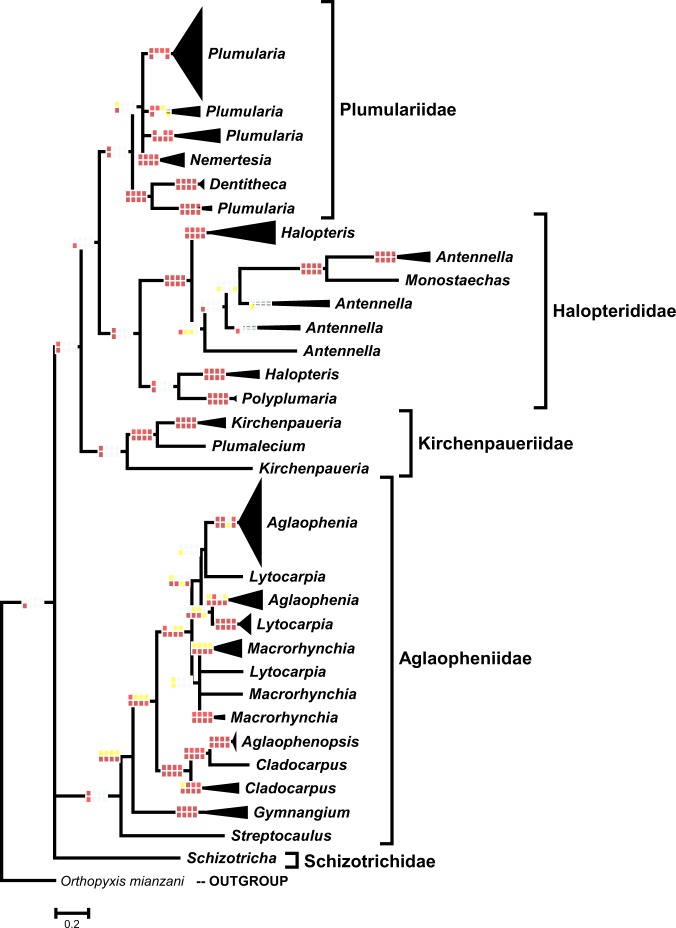
Phylogeny of Plumularioidea hydroids based on 16S+COI sequence data, without Gblocks manipulations, obtained with MrBayes. Nodes collapsed presented posterior probabilities below 70% for the phylogenetic analysis represented graphically. Rectangles on nodes show nodal support (for ML analyses: yellow – 50–74%; red – 75–94%; dark-red – 95–100%; for Bayesian analyses: yellow – 75–89%; red – 90–97%; dark-red – 98–100%) for the different phylogenetic analyses: first horizontal row – ML; second horizontal row – Bayesian; first vertical row – alignment with all families together with gaps; second vertical row – alignment without manipulations per family; third vertical row – alignment with some gaps removed, per family; fourth vertical row – alignment without gaps, per family. When nodal support is not indicated, all phylogenetic analyses achieved 95–100% of nodal support. See detailed composition of branches in Supplementary Fig. S2.

We analyzed in further detail phylogenetic relationships for 250 Plumularioidea specimens with concatenated COI and 16S sequence data. These analyses produced 209 unique sequence types, of 66 nominal species that belonged to 17 genera. Figure 4 and Supplementary Fig. S2 illustrate the hypothesized phylogenetic relationships among those taxa, based on 275 base-pairs (bp) of COI complemented with 533 bp of 16S.

We obtained mostly similar results (differences noted in the following subsections) between the phylogenetic hypotheses generated by the alignments with combined 16S and COI sequence data (Fig. 4; Supplementary Fig. S2) and the alignments with only 16S sequence data (Fig. 3; Supplementary Fig. S1). Additionally, we found a high level of coherence between morphological and molecular divergences of taxa.

### Phylogenetic relations among families

Our results are in part concordant with previous studies^6,16,21,38,40^, which recovered as monophyletic the five Plumularioidea families herein analyzed: Plumulariidae, Aglaopheniidae, Kirchenpaueriidae, Halopterididae and Schizotrichidae. However, with the addition of more taxa, the present results disagree partially with some of these conclusions. First, *Kirchenpaueria bonnevieae*, a species thought to belong to the family Kirchenpaueriidae, clusters within the family Plumulariidae. Additionally, *Pseudoplumularia marocana*, classified as a member of Plumulariidae, clusters inside the family Halopterididae (Supplementary Fig. S1; Fig. 3). Therefore, the families Kirchenpaueriidae and Plumulariidae are polyphyletic and the family Halopterididae paraphyletic unless other taxonomic changes are made.

The inclusion of the genus *Pseudoplumaria* in the family Halopterididae, instead of Plumulariidae, may be reconciled with morphology if the large “mamelons” characteristic of that genus^47^ are instead considered as atrophied hydrothecae, thus conforming with the diagnosis of Halopterididae^34^. Furthermore, the presence of gonothecal nematothecae in *Pseudoplumaria*, a character present in the family Halopterididae and generally absent in the Plumulariidae^47^, further supports the transfer of *Pseudoplumaria* to the Halopterididae. In addition, *Pseudoplumaria* also resembles morphologically the halopteridid genus *Polyplumaria*^47^ (pers. obs.), which is its sister group in the 16S phylogeny (Fig. 3; Supplementary Fig. S1).

In contrast, the inclusion of *Kirchenpaueria bonnevieae* within the cluster of Plumulariidae could represent morphological convergence. *Kirchenpaueria bonnevieae* has a single nematotheca above the hydrotheca (per internode) and has monothalamic nematothecae, characters considered exclusive to the family Kirchenpaueriidae. All other known Plumulariidae members, possess bithalamic nematothecae and paired lateral nematothecae.

The family Aglaopheniidae clusters with members of *Hydrodendron* (results not shown; but see Maronna *et al*.^40^), although support is low. Because available 16 S sequences of this genus were extremely divergent from the rest of Aglaopheniidae, we excluded these from the present analyses. In general agreement with previous phylogenetic works^6,16,21,38,40^, Aglaopheniidae (plus *Hydrodendron* spp.) are sister to an evolutionary branch called Plumulariida (*sensu* Maronna *et al*.^40^) that consists of the family Schizotrichidae as sister to the Kirchenpaueriidae, Halopteriidae and Plumulariidae (Fig. 3). However, our Bayesian analysis of the COI+16S dataset (Fig. 4) suggests Schizotrichidae is sister to the Aglaopheniidae, and both clades sister to the remaining Plumularioidea (as suggested, but poorly supported by Peña Cantero *et al*.^21^). We favor the “Plumulariida” result of Maronna *et al*.^40^ because that study used more conserved markers. Although not entirely conclusive, because nodal support was not significant for most phylogenetic analyses, some results suggest the Plumulariidae *s*.*l*. and Halopterididae *s*.*l*. could be sister groups, and this clade sister to the family Kirchenpaueriidae *s*.*s*. (Fig. 3).

As a note, recently Choong & Calder^48^ erected the family Plumaleciidae to accommodate *Plumalecium plumularioides*, but our results (Figs 3 and 4) suggest that this taxon may cluster within Kirchenpaueriidae. However, our specimen (USNM 1081744) identified as *Plumalecium* cf. *plumularioides* and collected from deep waters, relatively close to the type locality (i.e. shallow waters of Alaska) of the species, does not exhibit nematothecae and may differ by the presence of a nematophore in the apophyses. If that specimen represents a different species, it should nevertheless be closely related to *Plumalecium plumularioides*, raising some doubt about the validity of the family Plumaleciidae.

### Phylogenetic relations among genera

The great majority of the Plumularioidea genera represented in our analyses are polyphyletic (Figs 3 and 4; Supplementary Figs S1 and S2). Of the 21 nominal genera represented in our analyses, we recovered only nine monophyletic taxa, namely: *Taxella* and *Gymnangium* (Aglaopheniidae); *Schizotricha* (Schizotrichidae); *Oswaldella* and *Pycnotheca* (Kirchenpaueriidae); *Polyplumaria* and *Pseudoplumaria* (Halopterididae); *Nemertesia* and *Monotheca* (Plumulariidae). The genus *Gymnangium* was previously reported to be polyphyletic^6,18,23,25^. Consequently the genus *Taxella* was resurrected^25^ to provide monophyly to the genus *Gymnangium* (Fig. 3). The remaining genera appearing as monophyletic in our analyses (Fig. 3; Supplementary Fig. S1) are represented by only one (in which case monophyly was not tested) or only a few species.

The COI+16S phylogenetic hypotheses (Fig. 4; Supplementary Fig. S2) confirmed polyphyly of genera of the Aglaopheniidae (*Cladocarpus*, *Lytocarpia*, *Macrorhynchia*, *Aglaophenia*), Plumulariidae (*Plumularia*) and Halopterididae (*Halopteris* and *Antennella*). Other polyphyletic genera highlighted with 16S data were underrepresented or not represented in this analysis.

#### Family Aglaopheniidae

Five of the seven nominal genera of the Aglaopheniidae, namely: *Aglaophenia*, *Lytocarpia*, *Macrorhynchia*, *Aglaophenopsis*, *Cladocarpus* and *Streptocaulus*, are not monophyletic (Supplementary Fig. S1). Uniquely the genera *Taxella* and *Gymnangium* are monophyletic (Supplementary Fig. S1; see above). This finding supports that the morphology of the reproductive structures^34^ alone fails to be diagnostic of the Aglaopheniidae.

The genera *Streptocaulus*, *Aglaophenopsis* and *Macrorhynchia* are paraphyletic. The cluster of *Streptocaulus* includes *Cladocarpus bocki*; the clade of *Aglaophenopsis* includes *Cladocarpus carinatus*, and the branch with multiple lineages of *Macrorhynchia* includes *Lytocarpia nigra* (as well as *Taxella* spp.). The genera *Cladocarpus*, *Lytocarpia* and *Aglaophenia* are polyphyletic, a result partially revealed for the last two genera by Moura *et al*.^18^ and Postaire *et al*.^23^ (Figs 3 and 4; Supplementary Figs S1 and S2).

The nominal genus *Cladocarpus* comprises a main clade containing a branch with *C*. *paradiseus*, *C*. *paraformosus*, *C*. *integer* and *C*. *sigma*, which is sister to a branch containing *Aglaophenopsis* species and *Cladocarpus carinatus* (sister to *Aglaophenopsis cartieri*). In a distant phylogenetic position, we find *Cladocarpus bocki* clustered amidst *Streptocaulus* lineages (Figs. 3 and 4; Supplementary Figs S1 and S2).

Multiple lineages of *Lytocarpia* occur in distant phylogenetic positions clustered with taxa of other genera. For example, *Lytocarpia nigra* clusters amidst *Macrorhynchia* lineages, *L*. *canepa* clusters with *Aglaophenia sinuosa*, *L*. *brevirostris* clusters with *A*. *latecarinata*, and *L*. *delicatula* clusters in a clade with other *Aglaophenia* species (e.g., *A*. *trifida*, *A lophocarpa*, *A*. *pluma* complex) (Figs 3 and 4; Supplementary Figs S1 and S2).

The majority of the *Aglaophenia* species represented cluster together, excepting *A*. *sinuosa*, *A*. *latecarinata*, *A*. *rhynchocarpa*, *A*. *cupressina* and possibly *A*. *postdentata*. The phylogenetic associations of the first two species were mentioned above. While the phylogenetic placement of *A*. *rhynchocarpa*, *A*. *cupressina* and *A*. *postdentata* are somewhat uncertain with 16S data, these species do not cluster with the other *Aglaophenia* species represented (Fig. 3; Supplementary Fig. S1). Supplementary Fig. S2 further highlights the clustering of *A*. *rhynchocarpa* with *Lytocarpia myriophyllum*, and of *L*. *brevirostris* with *A*. *latecarinata*.

The Aglaopheniidae comprises four distinct and well-supported main clades (Figs 3 and 4; Supplementary Figs S1 and S2; result somewhat noted and corroborated by Moura *et al*.^18^ and Postaire *et al*.^23,24^). These include the following taxa: clade (1) *Streptocaulus* species plus *Cladocarpus bocki*; clade (2) *Gymnangium* species; clade (3) *Aglaophenopsis* and *Cladocarpus* species (excluding *C*. *bocki*); clade (4) *Aglaophenia*, *Macrorhynchia*, *Lytocarpia* and *Taxella* species. “Clade 1” seems the outgroup to all the other Aglaopheniidae represented; “clade 2” may be sister to a cluster containing “clade 3” and “clade 4” (Figs 3 and 4; Supplementary Figs S1 and S2). One could interpret these four main clades as distinct genera, a decision that would simplify the confusion related to the taxonomic categorizations in this family^28,47,49–54^. But, diagnostic features for these four clades are not readily apparent, although they may exist.

#### Family Schizotrichidae

The *Schizotrichidae* was created recently to accommodate exclusively the genus *Schizotricha* that was transferred from the family Halopterididae^21^.

*Schizotricha profunda*, a species collected from deep waters off Florida, was recovered as sister to a clade containing close haplotypes of Antarctic *Schizotricha* species. Despite the reasonably high genetic divergence (“P” distance of 15–22%) between these clades, the genus *Schizotricha* is monophyletic (Supplementary Fig. S1).

#### Family Kirchenpaueriidae

Four nominal genera of the Kirchenpaeuriidae *s*.*l*. are represented in the present data: *Kirchenpaueria*, *Pycnotheca*,*Oswaldella* and *Plumalecium*. Of these, the genus *Kirchenpaueria* is shown to be polyphyletic (Figs 3 and 4; Supplementary Figs S1 and S2).

We recovered three main clades of *Kirchenpaueria s*.*l*. (Fig. 3; Supplementary Fig S1), which could provide the basis for establishing distinct genera. “*Kirchenpaueria” bonnevieae*, as already noted, belongs to the Plumulariidae. The clade containing *Kirchenpaueria pinnata* (the type species of the genus) and the possible conspecific *K*. *similis* (Moura *et al*.^6^; present study), without doubt corresponds to the genus *Kirchenpaueria*. *Plumalecium* (cf.) *plumularioides*, a species until recently considered as a member of the genus *Kirchenpaueria*, together with haplotypes of the genus *Oswaldella* form a clade sister to a branch containing *Pycnotheca mirabilis* and *Kirchenpaueria pinnata*. The sister group to these Kirchenpaueriidae taxa is *Kirchenpaueria halecioides* (Supplementary Fig. S1). *Kirchenpaueria halecioides* is actually the type species of the genus *Ventromma* (Stechow)^55^, to which this species has been sometimes assigned until recently^33,34,56^. The resurrection of that genus, therefore, would seem well advised. However, *Plumalecium plumularioides* presents the diagnostic characters of the genus *Ventromma*^34^, but clusters in a divergent clade as a sister group to specimens of *Oswaldella* (Supplementary Fig. S1).

#### Family Halopterididae

*Antennella*, *Halopteris* and *Monostaechas*, the most speciose genera of the family Halopterididae^34^, are clearly polyphyletic (Figs 3 and 4; Supplementary Figs S1 and S2). The genera *Pseudoplumaria* and *Polyplumaria* are only represented by a single nominal species. Genera of the family Halopterididae are classified according to the growth form of colonies^31^, however, that criterion clearly fails to be diagnostic of natural groups within Halopterididae.

Three well-supported main clades are recovered within the Halopterididae (Figs 3 and 4; Supplementary Figs S1 and S2). One clade comprises *Pseudoplumaria* and *Polyplumaria*, which are genetically close sister groups (Fig. 3; Supplementary Fig. S1; 0.7–2.6% of “P” distance) sharing many morphological similarities^47^ (pers. obs.). Another main clade, possibly sister to the previously mentioned relationship (albeit without great nodal support in some analyses), includes the nominal species *Antennella* “*secundaria*” (“lineage 1”), *Halopteris minuta*, *H*. *schucherti*, *H*. *violae*, *H*. *diaphana* and *H*. *tenella* (Supplementary Figs S1 and S2). The third main branch of the Halopterididae includes other taxa identifiable as belonging to three nominal genera that are not monophyletic: *Halopteris* (nominal species: *H*. *vervoorti*, *H*. *sibogae*, *H*. *alternata*, *H*. *carinata*, *H*. *liechternii*, *H*. *geminata*, *H*. *catharina*); *Antennella* (nominal species: *A*. *secundaria*, *A*. *kiwiana*, *A*. *siliquosa*, *A*. *ansini*, *A*. *confusa*, *A*. *similis*), and *Monostaechas* (nominal species: *M*. *quadridens*) (Supplementary Figs S1 and S2). Complementing analyses (Supplementary Fig. S1), further suggest that within the “third main branch”, the clade containing *Halopteris sp*., *H*. *vervoorti*, *H*. *sibogae*, *H*. *alternata*, *H*. *carinata* and *H*. *liechterniii*, is sister to a clade containing *A*. *secundaria* (lineages 2–11), *M*. *quadridens*, *A*. *confusa*, *A*. *similis*, *H*. *geminata* and *H*. *catharina*.

#### Family Plumulariidae

Two of the four nominal genera of Plumulariidae, namely *Plumularia* (Figs 3 and 4; Supplementary Figs S1 and S2) and *Dentitheca* (Fig. 3; Supplementary Fig. S1), were not shown to be monophyletic. The genera *Nemertesia* and *Monotheca* are so far verified as monophyletic (Figs 3 and 4; Supplementary Figs S1 and S2). However, we note that the *Monotheca* is not always considered a valid taxon and these species are often classified as *Plumularia* (e.g., WoRMS^27^).

The clade corresponding to the true genus *Plumularia* contains the nominal species *Plumularia setacea* (the type species of the genus), *P*. *strictocarpa*, *P*. *virginiae*, *P*. *duseni*, *P*. *lagenifera*, *P*. *gaimardi*, *P*. *warreni*, *P*. *setaceoides*, *P*. *filicaulis* and *P*. cf. *hyalina* (which has morphological similarities with the genus *Monotheca*; see Watson^57^). It is not clear whether this “true *Plumularia*” clade is sister to a clade containing the genus *Nemertesia* or instead is sister to an evolutionary branch (possibly representing a cryptic genus) containing the nominal species *Plumularia mooreana*, *P*. *floridana* and *P*. *sinuosa* (Supplementary Figs S1 and S2).

Our results reveal the genus *Dentitheca* to be paraphyletic (Fig. 3; Supplementary Fig. S1) due to its inclusion amidst “*Plumularia*” species, namely “*Plumularia” habereri*, *“Plumularia elongata* and “*Plumularia” spiralis*. Although *“P”*. *habereri* and “*P*. *elongata*” have been occasionally considered as belonging to the genus *Dentitheca*^58,59^, *“P*.*” spiralis* has been unequivocally assigned to the genus *Plumularia*^60^. Thus, the morphological characters cited as distinctive to the genus *Denthitheca* (i.e., the presence of triangular lobes on the hydrothecal margin, according to Stechow^55^ and Bouillon *et al*.^34^) do not appear to be diagnostic. It is possible that loss of these characters and reversion to a more general *“Plumularia”*-type of morphology occurred in the lineage leading to *“Plumularia” spiralis*.

The genus *Monotheca* was recovered as monophyletic and appears to be the outgroup of the remaining Plumulariidae (Fig. 3; Supplementary Fig. S1). We included only four of the twelve recognized species of *Monotheca*, and therefore, cannot exclude the possibility that the morphologically diagnostic characters of this genus may not be valid (see Watson^57^). If we consider the represented haplotype of “*Plumularia* cf. *hyalina*” as a *Monotheca* species, the genus would be polyphyletic. Although we did side with the arguments of Watson^57^ and accepted that New Zealand material does not correspond to *Monotheca hyalina*, nor to the genus *Monotheca*. Nevertheless, due to the genetic distinctiveness of the *Monotheca* clade (Fig. 3; Supplementary Fig. S1), that includes its type species – *M*. *margaretta*, we recommend revalidation of the genus *Monotheca*.

### Species Delimitation Analyses

After the combination and interpretation of the species delimitation analyses undertaken, we propose 198 species of Plumularioidea hydroids (Supplementary Table S1, Supplementary Fig. S1). This number contrasts with the 123 nominal species plus the 17 unknown morphological species that we considered during the collection stage of this study. We have high confidence for the species delimitation of 125 hypothetical species, reasonable confidence for the splitting of 63 putative species and doubtful confidence of cladogenesis for ten species (Supplementary Table S1, Supplementary Fig. S1). For most of the doubtful scenarios, we suspect that species diversity may be higher, but in a few particular cases we suspect over-splitting of species. We present preliminary hypotheses about Plumularioidea species-level diversity, to be tested with further morphologic and genetic studies (cf. degrees of confidence for each putative species delimitation in Supplementary Table S1 and Supplementary Fig. S1).

#### Conspecificity of morphospecies

The species delimitation analyses did not differentiate in 16 instances between different morphotypes thought to represent distinct species (Supplementary Table S1, Supplementary Fig. S1). In these cases, there is either a need to synonymize taxa, based on the ineffectiveness of the established diagnostic morphological characters to characterize these taxa, low inter-specific sequence divergence, and/or incomplete lineage sorting (or hybridization). In Supplementary Text S1 we report and discuss each of these results.

#### Cryptic species diversity

The number of cryptic species revealed by the genetic data was high, especially for nominal species with larger sample sizes and wider geographic ranges (e.g. *Antennella secundaria* and *Plumularia setacea*) (Supplementary Table S1; Supplementary Figs S1 and S2). The species delimitation analyses proposed species subdivisions (i.e., cryptic diversity) for 13 nominal species of Aglaopheniidae, two of Kirchenpaueriidae *s*.*s*., six of Halopterididae and 10 of Plumulariidae (Supplementary Table S1; Supplementary Figs S1 and S2). Cryptic diversity is much more probable whenever the putative species of the same nominal species are not sister lineages, which was verified in the following taxa: *Aglaophenia acacia*, “*A*. *pluma* complex”, *Gymnangium speciosum*, “*G*. *allmani* and *G*. *sibogae* complex” (Aglaopheniidae), *Antennella secundaria*, *A*. *similis*, *Halopteris diaphana*, *H*. *alternata* (Halopterididae), *Plumularia strictocarpa*, *P*. *setacea*, *P*. *floridana*, *N*. *antennina* (Plumulariidae) (Supplementary Fig. S1). When putative species are sister lineages (e.g., *Monostaechas quadridens*) they could simply be indicative of intraspecific phylogeographic structuring, or incipient speciation. Indeed, high levels of population structuring and isolation by distance were revealed with microsatellite data, in two supposedly widely dispersed Indo-Pacific species of Aglaopheniidae^24,61,62^. These cases suggest that most of the cryptic diversity proposed by our species delimitation analyses (Supplementary Table S1 and Fig. S1) is highly probable, and in some cases, we suspect it could be even higher (cf. degrees of confidence for cladogenesis). Cryptic diversity is frequently suggested for lineages of a nominal species obviously separated by a physical barrier (e.g., American Continent) or long distances (e.g, both sides of the Atlantic). But cryptic diversity has also been suggested for individuals of a nominal species sampled in close spatial proximity. In Supplementary Text S2 we identify and discuss each case of putative cryptic diversity uncovered in this study.

### Unknown or new species

Ten morphotypes of Plumularioidea sampled for this study could not be assigned to any nominal species, due to their peculiar morphological characteristics and distinctive phylogenetic positions. There is a great chance these morphotypes correspond to undescribed (new) species. Interestingly, the possible new morphotypes identified were mainly collected from areas poorly sampled for taxonomic studies of marine hydroids, for example, Central America and deep-intermediate water depths. Supplementary Text S3 reports these potential new species.

### Taxonomic amendments

The present account intends to provide an overview of and draw attention to taxonomic problems of the superfamily Plumularioidea, one of the most common, biodiverse and well-studied groups of hydroids. As such, Plumularioidea likely serves as a model for what can be expected for other hydroid clades. Indeed, recent work on the clade Proboscoidea uncovered many of the same issues relating to species boundaries and diagnostic characters^63^. We are unable to make major taxonomic corrections here, because further sampling, and morphologic and phylogenetic analyses are still needed. However, considering the taxonomic inconsistencies in the groupings among Plumularioidea revealed by our data, as well as the putative timings of splitting of lineages^64^, we discuss what taxonomic amendments might be adopted in the future.

Concerning Aglaopheniidae, the four main clades identified could be interpreted as four different genera: (1) genus *Streptocaulus* Allman, 1883, that also contains “*Cladocarpus*” *bocki*. *Streptocaulus pulcherrimus*, the type-species of the genus, is not represented in our phylogeny, but the species of *Streptocaulus* that are represented form a single clade; (2) genus *Gymnangium* Hincks, 1874. The type species is represented - *Gymangium montagui*; (3) genus *Cladocarpus* Allman, 1874. This genus would subsume the genus *Aglaophenopsis* in agreement with Bouillon (1985). The type species of *Cladocarpus*, *Cladocarpus formosus* is not represented, but *Cladocarpus paraformus*, which is morphologically very similar^54^ clusters in that clade; 4) genus *Aglaophenia* Lamouroux, 1812. *Aglaophenia pluma*, the type-species, is represented. This grouping would imply that the genera *Macrorhynchia* Kirchenpauer, 1872, *Taxella* Allman, 1874 and *Lytocarpia* Kirchenpauer, 1872 would be synonymous to *Aglaophenia*, in accordance with the classification adopted before the sub-genus classification of these nominal genera by Kirchenpauer^65^.

For Kirchenpaeuriidae, an easy taxonomic decision would be to synonymize the four nominal genera included here into a single genus; *Kirchenpaueria* Jickeli, 1883 has priority. In fact, Bouillon *et al*.^34^ noted that the genera of this family are not clearly defined, presenting overlapping diagnoses.

For Halopteridiidae, we could interpret the three main clusters recovered within that family as three distinct genera: (1) genus *Polyplumaria* Sars, 1874, including *Pseudoplumaria* Ramil & Vervoort, 1992 and *Polyplumaria*; (2) a new genus to include the species *Halopteris minuta*, *H*. *schucherti*, *H*. *violae*, *H*. *tenella*, morphotypes similar to *H*. *diaphana*, as well as “*Antennella*”-like growth forms; (3) another genus including *Monostaechas quadridens* (the type species of *Monostaechas* Allman, 1877), nominal species of “*Antennella*” like *A*. *secundaria*, *A*. *siliquosa*, *A*. *kiwiana*, *A*. *ansini* and *A*. *confusa*, as well as members of the nominal genus *Halopteris* Allman, 1877 like *H*. *carinata* (the type species of the nominal genus *Halopteris*), *H*. *liechternii*, *H*. *polymorpha*, *H*. *geminata*, and morphotypes similar to *H*. *alternata*. However, no obvious morphological characters were identified to diagnose these hypothetical genera, and there is need for a broader sampling including other nominal taxa of Halopteriidae missing here (e.g., *Antennella gracilis* – the type species of *Antennella* Allman, 1877; genera *Antennellopsis* Jäderholm, 1896, *Corhiza* Millard, 1962, *Gattya* Allman, 1886, *Astrolabia* Naumov, 1955, *Calvinia* Nutting, 1900, *Pentatheca* Naumov, 1955, *Diplopteroides* Peña Cantero & Vervoort 1999, *Anarthroclada* Naumov, 1955 and *Nuditheca* Nutting, 1900).

For the Plumulariidae *s*.*l*., the following changes at the genus level could be adopted: (1) synonymy of the genera *Plumularia* Lamarck, 1816, *Nemertesia* Lamouroux, 1812 and *Dentitheca* Stechow, 1919, with the name *Plumularia* having priority; (2) the creation of a new genus to accommodate *Kirchenpaueria bonnevieae*; (3) validation of the genus *Monotheca* Nutting, 1900; (4) transference of the genus *Pseudoplumaria* Ramil & Vervoort, 1992 from the family Plumulariidae to the family Halopterididae.

The recently created family Plumaleciidae^48^ should not be considered valid.

At the taxonomic level of species, we verified some cases of probable conspecific morphotypes, suggesting either synonymies or simply inoperability of morphological diagnostic characters (Supplementary Fig. S1 and Text S1). Below we list these cases and taxonomic amendments that may be adopted for the following associations of morphospecies, listing the species name with priority first:

- *Taxella eximia* and *T. gracilicaulis*, probably synonymous;
- *Macrorhynchia phoenicea* and *M. spectabilis*, probably synonymous;
- *Aglaophenia pluma, A. octodonta* and *A. tubiformis*, possibly synonymous. However, the discovery of a cryptic clade within this complex complicates taxonomic rearrangements;
- *Aglaophenia lophocarpa* and *A. acacia*, possibly synonymous;
- *Aglaophenia struthionides* and *A. latirostris*, probably synonymous;
- *Gymnangium speciosum, G. sinuosum* and *G. allmani*. The morphologic diagnostic characters to differentiate these nominal species are likely useless, but the presence of cryptic clades in this complex prevents us from suggesting synonymies;
- *Kirchenpaueria pinnata* and *K. similis*, probably synonymous;
- *Plumularia micronema* and *Plumularia floridana*. Their morphologic diagnostic characters are not useful, but both species are probably valid, due to cryptic diversity found within *P. floridana*;
- *Nemertesia antennina* and *Nemertesia perrieri*, likely synonymous;
- *Monotheca posidoniae* and *Monotheca obliqua*. The diagnostic characters given to separate these nominal species are probably useless, but due to cryptic diversity found within *M. obliqua*, both species may be valid.

In contrast, the numerous cases of probable cryptic diversity highlighted (Supplementary Figs S1 and S2; Supplementary Text S2) within Aglaopheniidae, Kirchenpaueriidae, Plumulariidae and Halopterididae (Table 1) deserve further haplotype sampling, morphological studies and sequencing of nuclear markers. It is probable that around 34–41% of the richness of the Plumularioidea is cryptic (Table 1), awaiting formal description or resurrection of nominal species currently unaccepted.

**Table 1:** Number and proportion of new, nominal, cryptic and synonymous species in four Plumularioidea families.

	Aglaopheniidae		Kirchenpaueriidae		Halopterididae		Plumulariidae		Total	
	n	Proportion	n	Proportion	n	Proportion	n	Proportion	n	Proportion
cf. New species	10	12%	0	0%	4	8–9%	3	6%	17	8–9%
Nominal species	57	70%	10	91%	21	44–48%	29	53–56%	117	59–62%
Cryptic species	24 (−5)	24–30%	2	18%	24 (−3)	48–55%	26 (−5)	40–50%	76 (−13)	34–41%
Synonymies	10 (−4)	7–13%	1	9%	1	2%	3 (−2)	2–6%	15 (−5)	5–8%

Species delimination results we consider questionable are given in parentheses.

### Prospects for taxonomic work

Many diagnoses will have to be rewritten, some nominal species resurrected or synonymized, and new taxa described. Some of this work will likely take place on a piecemeal basis. But the ideal situation would be a comprehensive taxonomic revision in light of morphological biodiversity sampled from various depths and localities across the globe, integrated with molecular phylogenetic and species delimitation analyses. This taxonomic work will be complicated by the existence of many invalid or synonymized taxa (often insufficiently described), the loss or degradation of type specimens, and the general state of the taxonomic profession when detailed revisionary systematics works are discouraged as being too time consuming and of low short-term impact (as measured by citation indices).

To cope with the large number of nominal species placed in synonymy and high levels of cryptic diversity that often occur in close spatial proximity, we suggest that topotypes associated with molecular data be established for relevant nominal species with type material that is not amenable to genetic analysis. These topotypes should be morphologically similar to the original type specimens and collected from localities and depths close to original type localities. Ideally, we should also sequence DNA Barcodes of all types or topotypes of nominal species, including newly described species, to serve as reference for comparisons with morphologically similar taxa.

**Figure 5:**
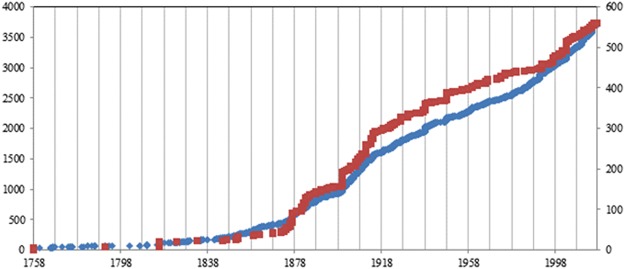
Progress of the number of taxonomic descriptions of the Hydrozoa (blue dots, left vertical axis) and of the superfamily Plumularioidea (red dots, right vertical axis) between the years 1758 and 2017. We considered only currently accepted species. Data source: WoRMS^27^.

Species descriptions of Plumularioidea hydroids, and of the class Hydrozoa (Fig. 5), increased drastically in the latter half of the 18^th^ century, reflecting the increased frequency of scientific expeditions; decreased slightly in the early-mid 20^th^ century, probably related to past World Wars; and quickened again from the 1990’s to the present.

In the past 259 years of “Linnaean” taxonomy, around two valid species of Plumularioidea (14 species of Hydrozoa) were described annually, on average. However, considering uniquely the past two decades, an average of five nominal species of Plumularioidea (36 species of Hydrozoa) have been described annually. Assuming a similar rate of species description as that of the past 20 years, and our result of around 10% of morphologically discernable morphospecies of Plumularioidea still undescribed (cf. Table 1), it would take approximately 11 years to describe the majority of the morphospecies of Hydrozoa or Plumularioidea. However, we would expect this to be an underestimate considering how much of the deep sea is still unexplored. The formal taxonomic description of cryptic diversity, which may represent around 34–41% of the overall diversity of the Plumularioidea (Table 1), will require significantly more time and effort, as it will have to involve genetic species-delineation analyses involving many samples, assessment of currently invalid taxa for possible resurrection, and formal redescriptions of species associated with newly designated topotypes.

## Conclusions

This work clearly illustrates that the more effort we put into comprehending the genetic diversity of hydroids, the more taxonomic pitfalls we find. Our results suggest the existence of a considerable number of errors in the diagnoses of families, genera and species. At the same time, our results present a realistic picture of the state of the systematics of Plumularoidea. Analyses of other hydrozoan groups uncovered many of the same issues relating to species boundaries and diagnostic characters^6–8,17,20,63^, thus supporting the assertion that the taxonomic problems highlighted here are likely to be persistent throughout Hydrozoa, and perhaps for Cnidaria as a whole.

At the family level, we uncovered inconsistencies in classification that are relatively easily addressed. *Pseudoplumaria marocana* and *Polyplumaria flabellata* are sister groups that likely belong to the family Halopterididae instead of Plumulariidae, whereas *Kirchenpaueria bonneviae* may belong to the Plumulariidae instead of Kirchenpaueriidae. *Plumalecium* cf. *plumularioides* (but with some differences as noted above) groups within the Kirchenpaueriidae, thus the recently erected family Plumaleciidae may not be valid.

The difficulty in dealing with Plumularioidea genera increases, as the majority of those genera represented were not monophyletic, namely: *Aglaophenia*, *Lytocarpia*, *Macrorhynchia*, *Aglaophenopsis*, *Cladocarpus* and *Streptocaulus* (Aglaopheniidae); *Kirchenpaueria* (Kirchenpaueriidae); *Antennella*, *Halopteris* and *Monostaechas* (Halopterididae); *Plumularia* and *Dentitheca* (Plumulariidae). This reveals that the morphology of the reproductive structures, and the mode of growth of colonies, fail to be diagnostic of genera for Aglaopheniidae and Halopterididae, respectively. Moreover, the dentition of the hydrothecal margin, the relative size of colonies and polysiphony/monosiphony of hidrocauli, may fail to diagnose genera of Plumulariidae. Although we do not make a formal recommendation, we are of the opinion that of instead of interpreting such taxonomic inconsistences as substantial underestimation of generic diversity (as Postaire *et al*.^23^ concluded for Aglaopheniidae), it could be more straight forward to synonymize most genera, instead of resurrecting or creating new genera (e.g. Ronowicz *et al*.^25^).

Our results suggest that around half of the species richness of the superfamily Plumularioidea is not formally recognized (Table 1). Of that portion, around 10% corresponds to discernible morphotypes that are as yet undescribed and 34–41% to cryptic species (Table 1). An incalculable proportion of the cryptic diversity corresponds to presently synonymized/unused species names with nomenclatural priority, which will complicate future taxonomic work. Additionally, further sampling from deep waters or in marine territories of developing countries or international jurisdiction, will likely uncover a higher proportion of undescribed taxa. On the other hand, around 5–8% of the currently accepted species represent probable synonymies (Table 1), and are thus invalid. The assessment and formal description of the real species diversity of the Plumularioidea, or of the Hydrozoa or Cnidaria as a whole, will take a significant, and probably incalculable, amount of time and multidisciplinary efforts.

It is frequently a great challenge to distinguish between intra and inter-specific boundaries, because morphological convergence and plasticity blur our ability to perceive many taxonomic relationships. Our work shows that many frequently employed characters/conditions are not particularly reliable for diagnosing a number of species. These include: presence/absence of ahydroathecate internodes in hydrocladia; robustness and color of colonies; elongation or not of hydrothecae; space between branches; space between hydrothecae; thickness or presence of intrathecal septum; presence/absence of ramification; size of mesial nematothecae; undulation of the hydrothecal margin; number of nematothecae on hydrocladial athecate internodes; and ecological specialization on certain algae.

To resolve hydrozoan systematics, monumental sampling efforts and sequencing of DNA markers of taxa and populations so far not represented are essential. In conjunction with molecular data, morphologic characters need to be re-inspected and contrasted with signal from molecular analyses in order to reform taxonomy and rewrite diagnoses. Nomination of topotypes directly associated with genetic data in situations of degraded or lost type specimens, is highly advisable in order to facilitate clarification of the taxonomy of these diverse, commonly encountered, ecologically important, and morphologically simple marine invertebrates.

Finally, the 16S marker provided good resolution for many phylogenetic relationships among Plumularioidea hydroids, especially after increasing representation of taxa. The addition of COI sequences (or other markers) helps to resolve further associations of lineages, but the community still needs to develop suitable primers to increase the efficiency with which this marker can be derived.

## Electronic supplementary material

Supplementary Information

Supplementary Table S1
