# Supplementary material for: Hundreds of genetic barcodes of the species-rich hydroid superfamily Plumularioidea (Cnidaria, Medusozoa) provide a guide toward more reliable taxonomy
Source: Sci Rep. 2018 Dec 20;8:17986. doi: 10.1038/s41598-018-35528-8 (PMC6301992; doi:10.1038/s41598-018-35528-8)
Supplement: Supplementary file 1 — Supplementary Information [file 41598_2018_35528_MOESM1_ESM.pdf]

Hundreds of genetic barcodes of the species-rich hydroid superfamily Plumularioidea (Cnidaria, Medusozoa) provide a guide toward more reliable taxonomy

Carlos J. Moura[1,2,3,6,*], Harilaos Lessios[2], Jorge Cortés[4], Martha S. Nizinski[3], John Reed[5], Ricardo S. Santos[1] & Allen G. Collins[3]

[1]MARE-IMAR-OKEANOS, Departamento de Oceanografia e Pescas, University of the Azores, Horta 9901-862, Portugal.

[2] Smithsonian Tropical Research Institute, Balboa 0843-03092, Panamá.

[3] National Systematics Laboratory, NOAA's National Marine Fisheries Service, Smithsonian National Museum of Natural History, Washington, DC 20560, USA.

[4] Centro de Investigación en Ciencias del Mar y Limnología (CIMAR), Universidad de Costa Rica, San Pedro, 11501-2060 San José, Costa Rica

[5]Harbor Branch Oceanographic Institute, Florida Atlantic University, Fort Pierce, Florida, 34946 USA

[6]Present Address: CBMA – Centre of Molecular and Environmental Biology, Department of Biology, University of Minho, Campus de Gualtar, 4710-057 Braga, Portugal.

* corresponding author: carlos.moura@mail.com

Supplementary text

Supplementary Text S1. Conspecificity between different morphotypes.

Within the family Aglaopheniidae, morphological species were not distinguished in ten situations: 1) *Macrorhynchia furcata* + *Macrorhynchia* sp1 + *Macrorhynchia* sp2 ; 2) *Taxella gracilicaulis* + *T. eximia* (lineage 2) ; 3) *Macrorhynchia phoenicea* + *M. sibogae* + *M. spectabilis* + *M. fulva* + *M. crestata*; 4) *A. tubiformis*, lineage 1 + *A. pluma* (lineage 1) ; 5) *Aglaophenia acacia* (lineage 1) + *A. elongata* ; 6) *Aglaophenia lophocarpa* + *A. acacia* (lineage 2) ; 7) *Aglaophenia latirostris* + *A. struthionides* ; 8) *Aglaophenia pluma* (lineage 2) + *A. tubiformis*, (lineage 2) + *A. octodonta* (lineage 3) + *A. harpago* ; 9) *Gymnangium speciosum* (lineage 1) + *G. sinuosum* ; 10) *Gymnangium speciosum* (lineage 2) + *G. allmani* (lineage 4). However, the degree of uncertainty was especially high in the first situation. Therefore, the morphotypes of *Macrorhynchia* sp1 and *Macrorhynchia* sp2 could possibly represent independent species. Additionally, taking into account the phylogenetic relationships revealed in Fig. 2, *Macrorhynchia phoenicea* and *M. spectabilis* are probably conspecific[24], but we have little evidence to assume that *M. sibogae*, *M. fulva* and *M. crestata* are synonymous. Similarly, we need further evidence to test whether *A. harpago* is conspecific with the *A. pluma* complex (clade with *Aglaophenia pluma* (lineage 2) + *A. tubiformis* (lineage 2) + *A. octodonta* (lineage 3)).

In the family Kirchenpaueriidae, the morphological species *Kirchenpaueria pinnata* (lineage 2) and *K. similis* are indistinguishable with 16S sequence data (Fig. 3); the Antarctic *Oswaldella* species represented could not be distinguished by the automated species delimitation methods. Interestingly, the five Antarctic *Oswaldella* species represented present very close 16S haplotypes (0-1% “P” distance), perhaps as a result of a radiation after a recent glacial age (as somewhat suggested by the phylogeny of Moura et al.[1]) and/or slower evolution of the 16S gene in these cold-water species.

In the family Halopterididae, the delineation methods could not distinguish, with high degree of confidence, between morphotypes of *Antennella similis* (lineage 1) and *A. secundaria* (lineage 5).

The species delimitation analyses did not differentiate nominal species of the family Plumulariidae in four situations: 1) *Plumularia setacea* (lineage 7) + *P. duseni* + *P. lagenifera* + *Plumularia virginiae*; 2) *Plumularia micronema* + *P. floridana* (lineage 4); 3) *Nemertesia antennina* + *Nemertesia perrieri* (as noted by Moura et al.[2]); 4) *Monotheca posidoniae* + *Monotheca obliqua* (lineage 1). However, especially in the first situation, the degree of confidence is not high enough to conclude conspecificity of the correspondent morphotypes/lineages.

Excluding the cases mentioned above where the degree of confidence is low, below we discuss the morphologic convergence, inoperability of diagnostic characters and/or possible synonymy between the following associations of nominal species:

- *Taxella eximia* and *T. gracilicaulis* - Aglaopheniidae. As noted by Ronowicz et al.[3], morphotypes of these nominal species are very similar and the supposedly diagnostic characters include: presence/absence of hinge-joints at the end of first segments of branches, robustness of colonies, the elongation of hydrothecae and the size and space between branches. Because the 16S haplotypes of these morphotypes do not cluster independently (Fig. 2), these nominal species may be conspecific[3,4], with *Taxella eximia* having nomenclatural priority.

- *Macrorhynchia phoenicea* and *M. spectabilis* - Aglaopheniidae. According to Di Camillo et al.[5] these morphotypes reproduce at similar time periods and are distinguished by the space between hydrothecae, and robustness and colors of colonies. These characters are probably plastic and influenced by abiotic conditions. Because haplotypes of these nominal species do not cluster independently, these taxa are thus probably synonymous[4] (present study), with the name *Macrorhynchia phoenicea* having priority.

- *Aglaophenia pluma*, *A. octodonta* and *A. tubiformis* - Aglaopheniidae. According to Svoboda & Cornelius[6] several features can differentiate these taxa, i.e., length of hydrothecae, thickness or presence of the intrathecal septum, distance between branches, and color of colonies. However, we disagree that these characters are useful for diagnosing these morphospecies, which might on the face of it suggest that the three species are synonymous under the name *A. pluma*. However, our data reveal two cryptic divergent clades for this complex of nominal species (one potentially also including *A. harpago*), and we are unable to assign names to either of these clades without further study.

- *Aglaophenia acacia* and *A. lophocarpa* – Aglaopheniidae. According to Svoboda & Cornelius[6] these nominal species are very similar and can only be differentiated by the presence/absence of ramification of colonies. Because no monophyletic cluster was obtained for these morphospecies, they are likely synonyms, having the name *A. lophocarpa* based on priority. Furthermore, the name *A. acacia* likely has been used erroneously in the identification of other taxa from the NE Atlantic or Mediterranean (e.g., *A. elongata*; cf. arguments in Supplementary Text S2).

- *Aglaophenia latirostris* and *A. struthionides* – Aglaopheniidae. These taxa are distinguished by the prominence or not of the mesial nematotheca[7], a character we observed to be quite variable even within a single colony in our material. Additionally, the small genetic distance between both morphotypes (0-0.7% "P" distance) and the output of the species delimitation analyses suggest synonymy of these taxa, with the name *A. struthionides* having priority. However, *A. latirostris* has been frequently reported from the NE Pacific[8-10], but Nutting[29] described it as originally collected from Brazil, a locality probably given inaccurately because it has not been collected by others in the Atlantic[11,12].

- *Gymnangium sinuosum*, *G. speciosum* and *G. allmani* – Aglaopheniidae. The first two of these nominal species differentiated by the undulation of the hydrothecal

margin[13,14], cluster tightly (1.7% "P" distance) (Fig. 2). Considering also the output of the species delimitation analyses, these taxa are probably conspecific, having the name *G. speciosum* based on priority. However, we found another divergent genetic clade (10.3% "P" distance) of *G. speciosum* that clusters tightly (0.3% "P" distance) with a cryptic haplotype of *G. allmani,* suggesting that the size of the mesial nematotheca also fails to be diagnostic, in this case to differentiate *G. allmani* from the other two nominal species. Nevertheless, the crypsis suggested either within *G. allmani* and *G. speciosum* complicates future intentions to clarify the taxonomic status of these nominal species plus *G. sinuosum.*

- *Kirchenpaueria pinnata* and *K. similis* – Kirchenpaueriidae. These nominal species differentiated by the presence/absence of intermediate athecate internodes in the hydrocladia, do not segregate into independent lineages (Fig. 3), supporting the opinion that these are conspecific[15,16], with the name *K. pinnata* having priority.

- *Antennella similis* and *A. secundaria* – Halopterididae. Lineages of both species ("lineage 1" and "lineage 5", respectively) cluster together tightly (0.7% "P" distance), suggesting that these clades are conspecific. Therefore, the diagnostic characters given by Galea[17] to differentiate these species do not seem to be valid, namely the "occurrence of very short hydrothecate segments ending immediately above the axillar nematotheca" and "the presence of an incomplete, basal, transverse node in the ahydrothecate segments". However, the finding of cryptic species, both within *Antennella similis* as well as *A. secundaria*, support the validity of these species, but also the need to define them unambiguously.

- *Plumularia micronema* and *P. floridana* – Plumulariidae. These nominal taxa supposedly differ by the presence/absence of athecate internodes between hydrothecate internodes[18], but a morphotype identifiable as *Plumularia micronema* seems conspecific with a cryptic lineage of *P. floridana* (lineage 4). Thus, the present diagnoses are not sufficient to differentiate these nominal species. Because *Plumularia floridana* Nutting, 1900 seems to present cryptic diversity (Supplementary Fig S1), *Plumularia micronema* Fraser, 1938 may be a valid species if it corresponds to a cryptic lineage of *P. floridana.*

- *Nemertesia antennina* and *Nemertesia perrieri* – Plumulariidae. These nominal species differ by the constant presence of two nematothecae on the athecate internodes (or two consecutive athecate internodes with one nematotheca)[16]. In the NE Atlantic and Mediterranean, the same 16S haplotype is shared by morphotypes with diagnostic characters of both nominal species[2] (Supplementary Fig S1). However, a cryptic lineage found in the Azores (collected near Terceira Island) may prove conspecific with *Nemertesia perrieri*, originally collected from the Canaries[19], supporting the validity of that species despite few differences based on the morphologic characters presently used to diagnose it.

- *Monotheca posidoniae* and *Monotheca obliqua* – Plumulariidae. The unique diagnostic character accepted to distinguish *M. posidoniae* is its obligatory growth on *Posidonia*[20,21]. Considering that the haplotypes of both nominal species represented diverge little in the Mediterranean (almost 0% "P" distance), our results support the invalidity of *Monotheca posidoniae*[20]. However, considering the present revelation of putative cryptic diversity within *M. obliqua*, and that the type locality of *M. obliqua* is in

the British Isles and that of *M. posidoniae* is in the Mediterranean, we do not discard the possibility that these are independent species in need of a reliable diagnoses.

Supplementary Text S2. Cryptic diversity.

- *Family Aglaopheniidae*

1) *Cladocarpus paradiseus* - two putative sister species (3.6-4.3% "P" distance), occurring in sympatry, in deep waters of Eastern Florida at similar depth ranges.

2) *Cladocarpus sigma* - two putative sister species (4% "P" distance), each from each side of the North Atlantic.

3) *Taxella eximia* - two putative sister species (3-5% "P" distance), one from the tropical West Pacific, the other from the SW Indian Ocean, which includes multiple clades of *T. eximia* clustered amidst morphotypes of *T. gracilicaulis*.

4) *Macrorhynchia philippina* - two putative (possibly sister) species (3.3-4% "P" distance), one with samples from the SW Indian Ocean and tropical W Pacific, the other presenting a circum-tropical and subtropical distribution.

5) *Lytocarpia brevirostris* - six putative sister species (0.7-7.9% "P" distance between species), four of these from the SW Indian Ocean, the remaining two from New Caledonia, clustering together amidst the others. Results of this species delineation analyses has some uncertainty associated; analyses of Postaire et al.[22] proposed four putative species within *L. brevirostris*, but Postaire et al.[4], using microsatellite data, suggested cryptic diversity was quite high for this species.

6) *Aglaophenia latecarinata* - two putative sister species (2-2.6% "P" distance), one present in the Caribbean, the other from Brazil.

7) *Aglaophenia trifida* - three putative sister species (1.3-3.6% "P" distance), occurring in close geographic proximity in the SW Caribbean.

8) *Aglaophenia pluma* complex (comprising *A. pluma*, *A. tubiformis*, *A. octodonta*) - three putative species (3-5% "P" distance between putative species), one represented by an Azorean clade sister to other putative species present in the Mediterranean and S Portugal; the third putative species (that also includes a monophyletic clade of *A. harpago*) includes samples from various locations in coastal waters of Europe and Macaronesia islands. Moura et al.[23] had already noted polyphyly of morphotypes of *A. pluma*, *A. tubiformis* and *A. octodonta* within the "*A. pluma* complex" clade containing *A. harpago*. However (cf. also Supplementary Fig S1), this study reveals the existence of another relatively distant (genetic) clade containing the other two putative species that also present the morphotypes of *A. pluma*, *A. tubiformis* and *A. octodonta* (noting that few samples were identified by A. Svoboda, who revised *Aglaophenia* species[24]). Adding further taxonomic complications within the "*Aglaophenia pluma* complex", its two main divergent clades occur in close geographic proximity.

9) *Aglaophenia acacia* – three putative species (2-2.6% "P" distance): one collected from the Italian littoral zone has the same haplotype as *A. elongata*; another collected from the coast of Galicia (and identified by A. Svoboda) clusters closely amidst clades of *A. lophocarpa*; and another collected from S Portugal presents no particular genetic relation with other species within a clade containing several *Aglaophenia* species. *Aglaophenia acacia* was originally described from deep waters of the Azores[25], later recorded in that archipelago at intermediate depths (cf. Cornelius[26]) and was posteriorly registered in the W Atlantic and European coasts from Brittany to the Canary Islands, including the eastern Mediterranean (cf. Cornelius[27]). Cornelius[27] already pointed out that *Aglaophenia lophocarpa* and *A. acacia* could be synonymous and our results support that suspicion (having the name *A. lophocarpa* based on priority), especially because the Azores were quite well sampled for this study. Additionally, we also show the name *Aglaophenia acacia* has been used erroneously to identify other taxa.

10) *Lytocarpia phyteuma* - six putative species clustering monophyletically (1-4.3% "P" distance between putative species), three of these are sisters in a tropical West Pacific clade, the other three are in a sister clade with Indian Ocean lineages. Postaire[22] revealed similar results, though slightly less inflated.

11) *Gymnangium speciosum* - two species (10% "P" distance), occurring in relative close proximity in the SW Caribbean Sea but presenting other species as sister groups (*G. sinuosum* and *G. allmani*, respectively).

12) *Gymnangium allmani* and *G. sibogae* complex - four putative species (7-9.6% "P" distance), two of these from the Indo-Pacific that are to be classified as *Gymnangium sibogae* after Galea & Di Camilo[28], the two other species identifiable as *G. allmani* are from the NW and NE Caribbean coast of Panama respectively. Although the latter two species are sister groups, one clusters more tightly with a morphotype identifiable as *Gymnangium speciosum*. The type locality of *Gymnangium allmani* is Brazil (59 m depth), thus one of the two haplotypes from the Caribbean represented is more likely to correspond to the true species. As suspected by Galea[17], the *G.* "*allmani*" known from the Indo-Pacific corresponds to a different species. Recently, Galea & Di Camillo[28] resurrected the species *G. sibogae* for the Indo-Pacific records of *G. allmani*, after morphological revision of Indonesian samples (type region). However, we have two Indo-Pacific lineages of *G. sibogae*/*allmani* represented, one from the SW Indian Ocean and another from New Caledonia, placed in different phylogenetic positions. Thus, we cannot be sure which lineage corresponds to the true nominal species, in this case *G. sibogae*.

- *Family* Kirchenpaueriidae

1) *Kirchenpaueria pinnata* - two putative sister species (2.6-3.6% "P" distance), one with samples from N California, the other with multiple lineages from the NE Atlantic. The NE Pacific clade likely represents a cryptic species (according to the type locality), that probably maintained trans-Arctic gene flow with the Atlantic counterparts

during the Pliocene and then likely became genetically isolated. Interestingly, *K. pinnata* seems to disperse effectively both in shallow and deep waters of the NE Atlantic.

2) *Kirchenpaueria halecioides* - two putative sister species (4-4.3% "P" distance), one with a wide distribution across distant areas of the Atlantic, the other corresponding to a sample collected in the Azores. This nominal species supposedly has a worldwide distribution in tropical, subtropical and temperate shallow waters[16]. We uncovered nine haplotypes for this nominal species, mostly collected from marinas or ports. A clear indication that this species notably takes advantage of boat traffic to disperse to distant locations, is that one of the haplotypes was collected in shallow waters of Argentina and in several marinas of mainland Portugal, Madeira and Azores, including from a ship-hull. It is also noteworthy that the specimens of *K. halecioides* collected from the Caribbean cluster together and apart from the others, but without great genetic distance. This is likely a reflection of adaptability of haplotypes to specific seawater temperatures. The (possible) cryptic haplotype collected from the port of Horta (Azores) diverges considerably from the other haplotypes sampled (although it clusters together) and may represent an exotic (or cryptic endemic) species in the Azores.

- *Family* Halopterididae

1) *Polyplumaria flabellata* - two putative sister species (1-2% "P" distance), one with a single haplotype shared between the Gulf of Cadiz and Bay of Biscay, the other containing sixteen haplotypes found exclusively in the Azores region. *Polyplumaria flabellata* is an eastern Atlantic species with a wide bathymetric distribution[16]; type locality is in deep waters off Norway[29].

2) *Antennella secundaria* - eleven putative species (1-23% "P" distance), one from central W Africa in a clade with *Halopteris minuta*, *H. schucherti*, *H. violae*, *H. diaphana* and *H. tenella*; the remaining ten putative species in the other main divergent clade of Halopterididade. These ten putative species include: one collected from the Caribbean coast of Panama ("lineage 2"); three sister cryptic lineages sister to *A. confusa* (one in shallow and deep waters of Europe and shallow waters of the Azores and Gorringe Bank; one from shallow waters of Madeira sister to the latter lineage, and one from the W Caribbean); two sister putative species from the central E Pacific; one from Indonesia; another from Madeira (in the same subclade of the last lineage); one present in deep waters of the Gulf of Cadiz and W Mediterranean sister to *H. catharina*, and another from the Pacific side of Costa Rica sister to a morphotype of *Monostaechas*. Part of such extensive cryptic diversity is further highlighted in Supplementary Fig S1. *Antennella secundaria* was originally described from the Mediterranean, being considered cosmopolitan with preference for warmer and temperate waters, and supposedly dispersing from littoral to bathyal depths[16,30]. Great phylogeographic structure was found within this nominal species, and we do not identify haplotypes or putative species presenting considerably large geographic ranges. Additionally, we found multiple cryptic lineages of *A. secundaria* sharing the same geographical area (like the Caribbean, Central East Pacific or NE Atlantic), which will complicate future taxonomic arrangements

within this nominal species. Nevertheless, although not many samples from the Mediterranean (type locality) were included, it is notorious that the unique shallow-water haplotype of this species available from the Mediterranean fits into a main clade containing *A. secundaria* morphotypes collected from NE Atlantic (including both shallow and deep waters, and Madeira and Azores) and also the Caribbean. Whatsoever, is remarkable the genetic segregation of sub-branches of that main clade between geographical regions (possibly indicative of crypsis). In addition, whilst *A. secundaria* ("lineage 3") may effectively disperse both in shallow and in deep waters, it is also noteworthy that we found a cryptic (somewhat phylogenetically unrelated) clade with samples collected from the Gulf of Cadiz and Alboran Sea that may be exclusively present in deep waters.

3) *Antennela similis* - three putative species in different clusters of species (5.6-17.2% "P" distance), one from the Caribbean side of Panama clusters tightly with a morphotype of *A. secundaria* from Belize, another from Florida clusters with one morphotype of *A. secundaria* from Indonesia, and the third found either in central W Africa and in the Caribbean off Panama (with one haplotype found on both sides of the Atlantic) sharing a same clade with the previous putative species conjunctly with a morphotype of *A. secundaria* from Madeira. Galea[17] described and erected *Antennela similis*, *A. peculiaris* and *A. tubitheca*, with type localities in the Caribbean, after noting 'deviant' morphotypes that do not correspond to *A. secundaria*. Although our results suggest those three taxa are independent from *A. secundaria*, these species possess many morphological similarities not discussed or diagnosed by Galea[17], and no DNA Barcodes were provided for the correspondent type material (that was fixed in 4% formalin). Therefore, we cannot ensure which lineage identified as *Antennela similis* or *A. peculiaris* is cryptic or not. This example highlights the importance of depositing/selecting type specimens (or parts) preserved in such a way that allows for molecular studies (as noted quite some time ago by Dayrat[31]).

4) *Halopteris diaphana* - four putative species (2-7.6% "P" distance): one present in the central E Pacific, two as sister clades with samples from Madeira Island, and a fourth species with samples present in the Mediterranean, Azores, Madeira and mainland Portugal that form an outgroup to a major clade containing the previous putative species and also *H. tenella* and *H. violae*, implying paraphyly. *Halopteris diaphana*, originally described from the Mediterranean[32], is considered to have a circumtropical distribution in shallow waters[16]. Although, Schuchert[33] considered records outside Europe doubtful mainly due to possible confusion with other similar species, namely *H. tenella*, *H. alternata*, *H. platygonotheca* and *Antennella siliquosa*. Morphological identifications of the specimens herein identified as *H. diaphana* were re-checked, supporting the conclusion that cryptic diversity within this taxon is highly probable. The *H. diaphana* – "lineage 4", which contains Mediterranean specimens, most probably corresponds to the "true" *H. diaphana*.

5) *Halopteris alternata* - five putative species (2-5% "P" distance): one found in NW Panama sister to *Halopteris polymorpha*, one from Belize, another with representatives in central W Africa and central E Pacific, one present in Miami and Belize, and a fifth more distantly related clade exhibiting a broad distribution including

the Caribbean, Brazil, central W Africa, Pacific side of Costa Rica and Madeira archipelago. *Halopteris alternata* is considered a shallow-water species with amphi-Atlantic distribution very rarely seen in the Pacific, with type locality in the West Indies – Caribbean[33,34,35]. Four of the five hypothetical species proposed by our species delineation analyses contain samples from the Caribbean (type region), complicating attempts to show correspondence with the "true" *H. alternata*. It is also noteworthy, in the clade of "*Halopteris alternata* - lineage 5", of one haplotype occurring simultaneously in the Caribbean, Brazil and W Africa, and another 16S haplotype present in W Africa (São Tomé and Principe and Savage Islands) and Caribbean coast of Panama. The wide dispersal of these haplotypes was likely due to rafting capabilities either on artificial and/or natural substrates[1]. Perhaps equatorial currents could explain the trans-Atlantic distribution of these two haplotypes. Although, sharing of a same 16S haplotype between the Pacific and Atlantic of Central America, suggests either punctual low molecular evolution of the 16S marker for this species, or perhaps (more probably) human-mediated dispersal[1].

6) *Monostaechas quadridens* - five putative species (1.3-7.3% "P" distance) clustering in a single clade: one from the Pacific of Central America, one from Brazil, two from the Caribbean, and another from Madeira, which is sister to the W Atlantic lineages. *Monostaechas quadridens* is a species mainly found in tropical, subtropical and temperate shallow waters of the Atlantic and Indo-Pacific[16,30,36], originally described from a specimen found on floating material in a harbor of South Carolina (SE USA). The represented haplotypes cluster monophyletically, and it is interesting that the lineages segregate according to geographical region. Therefore, one of the two putative species with Caribbean clades is most likely to correspond to the true *M. quadridens*.

- *Family* Plumulariidae

1) *Plumularia strictocarpa* - three putative species (3.3-6.3% "P" distance) possibly not clustering together: one present in the Maldives and SW Indian Ocean, one in Brazil and perhaps Caribbean too, another present in Moorea. *Plumularia strictocarpa* is morphologically similar to *P. setacea*, with type locality in the Moluccas, i.e. by the transition between the Indian and Pacific Oceans. Therefore, excluding the W Atlantic lineage that is clearly separate phylogenetic species, it is difficult to be sure which of the other Indo-Pacific clades may be the "true" *P. strictocarpa*.

2) *Plumularia setacea* - fourteen putative cryptic species (1.3-6.6% "P" distance), found in a well-supported (and highly-sampled) clade containing the nominal species *Plumularia setacea* and the morphologically similar species: *Plumularia strictocarpa*, *P. virginae*, *P. duseni*, *P. lagenifera*, *P. gaimardi* and *P. warreni*. The putative species within *P. setacea* are: one from the Caribbean of Panama; one present in the Mediterranean,mainland Portugal and the Azores; another from the Azores; two sisters from the SW Indian Ocean; one frequent on European coasts and across Macaronesia islands including the Gorringe seamount peaks; one putative species (including also the nominal species *P. virginiae*, *P. duseni* and *P. lagenifera*) with anti-tropical distribution along the Pacific coast of the American continent, and also present

in Argentina and New Zealand; another from South Africa that seems to cluster with *P. gaimardi* also from S. Africa (Supplementary Fig S1); one from the SW Caribbean clustered with a morphotype of *P. strictocarpa* from Brazil; one with representatives from Madeira; another with representatives from the Caribbean and tropical W Africa; one from the Pacific side of Panama (possibly) sister to other cryptic species from the Caribbean; and another found in Florida. *Plumularia setacea*, with type locality in the U.K., was assumed to be a cosmopolitan species, with no records from the Antarctic region to date[16]. Our results complement previous findings of Moura et al.[2] and Schuchert[37] that revealed extremely high levels of cryptic diversity within *Plumularia setacea*. The only putative species recorded near the type locality was that named as "lineage 6"; thus it likely corresponds to the true *Plumularia setacea*. That lineage seems the more frequently encountered across the NE Atlantic and Mediterranean coasts, being distributed both in shallow and deep waters. However, to complicate future taxonomic assignments and amendments, three other putative species are also present in shallow waters of the NE Atlantic where the (apparent) true *P. setacea*, i.e. "lineage 6", co-occurs. Additionally, the great phenotypic variability exhibited between the haplotypes of the putative species "*Plumularia setacea* – lineage 7" highlights the difficulty of differentiating between intra- and inter-species diversity.

3) *Plumularia setaceoides* - two sister putative species (2% "P" distance), occurring in relatively close geographic proximity, i.e. Australia and New Zealand.

4) *Nemertesia antennina* - three putative species (1.3-4% "P" distance): one with samples from coastal waters of Europe representing the true species, a second with multiple deep-water samples from the Azores, Gulf of Cadiz, Bay of Biscay and W Mediterranean, and another with deep-water samples from the W Atlantic and Gulf of Cadiz. It is noteworthy that the last mentioned lineage (putative cryptic species) has haplotypes in deep waters from both the western and eastern N Atlantic, denoting an ancient trans-Atlantic genetic connection across deep waters of the North Atlantic probably up to the Pliocene[1]. *Nemertesia antennina* was considered a cosmopolitan species[16], but as Moura et al.[2] demonstrated, it seems to be restricted to the shallow waters of the NE Atlantic and Mediterranean. Azorean and deep-water specimens once identified as *N. antennina* correspond to different cryptic species, and as suspected by Moura et al.[2] and somewhat suggested by the present species delineation analyses, cryptic taxa of *Nemertesia* "*antennina*" may be quite high. High cryptic diversity is possibly due to a combination of factors, like reduced populations sizes, delicate colony growth forms and local characteristics of deep-sea environments.

5) *Nemertesia belini* - two putative sister species (4.3-4.6%), one from the W Mediterranean, the other present in the Azores and Madeira (Moura et al.[2]; present study). The Mediterranean lineage is likely the cryptic one, because the type locality of *N. belini* is in the Azores.

6) *Plumularia floridana* - four putative cryptic species (1-3.3% "P" distance): two present in the W Caribbean, the other two in the tropical E Pacific. Within a clade of a cryptic Pacific lineage, some internal lineages, also identifiable as *Plumularia micronema* and *P.* cf. *propinqua*/*micronema*, that share obvious morphological similarities with *P. floridana*. Either these species demonstrate morphological plasticity

or these results provide evidence for the lack of diagnostic characters to characterize cryptic species within the nominal species *Plumularia floridana*.

7) *Monotheca margaretta* - three putative species (2.6-13.6% “P” distance): one from Madeira, sister to other with lineages in the Caribbean, Mediterranean and Macaronesia Islands, and another from Brazil clustering with morphotypes of *Monotheca posidoniae* and *Monotheca obliqua*. *Monotheca margaretta* was originally described from the Caribbean[7], so possibly the true species may refer to the clade (“lineage 2”) sampled from that region as well as from the NE Atlantic and Mediterranean. The lineage sampled from Brazil is likely a cryptic species.

8) *Monotheca obliqua* - three putative (possibly sister) species (2.6-3.3% “P” distance): one from the Mediterranean, one sampled in the Caribbean, and one present off mainland Portugal and the Azores. Because *M. obliqua* was originally described from the U.K.[38], the clade present in mainland Portugal or the one from the Mediterranean are the more probable candidates to correspond directly to the type material of that species. If the *M. obliqua* lineage from the Mediterranean, which contains the nominal species *Monotheca posidoniae*, is confirmed as an independent species in the future, this may validate the distinctiveness of *M. posidoniae,* but it would not be possible to diagnose it based on the substrate preference on algae.

Supplementary Text S3. Species unknown herein mentioned for the first time.

- *Family Aglaopheniidae*

1) *Macrorhynchia* sp1 - collected off Georgia, 67 m, in the SE USA; 2) *Macrorhynchia* sp2 – collected in the Glovers atoll, at 30-17 m, in Belize; 3) *Macrorhynchia* sp3 – collected near Carrie Bow Cay, at 40-20 m, in Belize; 4) cf. *Macrorhynchia* sp. – sampled at La Cornuda, at 20-10 m, in the W coast of Costa Rica, Pacific; 5) cf. *Gymangium* sp. – collected in St Johns, at 27-178 m, in Antigua (Caribbean).

- Family Halopterididae

1) *Antennella* sp. - collected at 27-15 m depth, at Isla Tambor in the Caribbean coast of Panama; 2) *Monostaechas* sp. - collected from shallow waters of the SW coast of Nicaragua, Pacific; 3) “*Monostaechas*” sp. - collected from shallow waters off Panama City, Pacific.

- *Family Plumulariidae*

1) *Plumularia* sp1 – collected from deep waters of the SE USA; 2) *Plumularia* sp2 – collected from deep waters of the Bahamas; 3) *Nemertesia* sp. – collected from deep waters of Florida.

REFERENCES

1. Moura, C. J., Collins, A. G., Santos, R. S., Lessios, H. Phylogeography of Plumularioidea hydroids: predominantly westward colonisations across major oceanic barriers. (In prep.)
2. Moura, C. J., Cunha, M. R., Porteiro, F. M., Yesson, C. & Rogers, A. D. Evolution of *Nemertesia* hydroids (Cnidaria: Hydrozoa, Plumulariidae) from the shallow and deep waters of the NE Atlantic and western Mediterranean. *Zool. Scr.* **41**, 79–96 (2012b).
3. Ronowicz, M., Boissin, E., Postaire, B., Bourmaud, C. A. F., Gravier-Bonnet, N. & Schuchert, P. Modern alongside traditional taxonomy: Integrative systematics of the genera *Gymnangium* Hincks, 1874 and *Taxella* Allman, 1874 (Hydrozoa, Aglaopheniidae). *PLoS ONE* **12(4)**, e0174244 (2017).
4. Postaire, B., Magalon, H., Bourmaud, C. A. F. & Bruggemann, J. H. Molecular species delimitation methods and population genetics data reveal extensive lineage diversity and cryptic species in Aglaopheniidae (Hydrozoa). *Mol. Phylogenet. Evol.* **105**, 36–49 (2016).
5. Di Camillo, C. G., Puce, S. & Bavestrello, G. *Macrorhynchia* species (Cnidaria: Hydrozoa) from the Bunaken Marine Park (North Sulawesi, Indonesia) with a description of two new species. *Ital. J. Zool.* **76 (2)**, 208–228 (2009).
6. Svoboda, A. & Cornelius, P.F.S. The European and Mediterranean species of *Aglaophenia* (Cnidaria: Hydrozoa). *Zool. Verh. Leiden* **274**, 1–72 (1991).
7. Nutting, C. C. American hydroids. Part I. The Plumularidae. *Spec. Bull. U. S. Nat. Mus.* **4 (1)**, 1-285 (1900).
8. Fraser, C. M. Hydroids of the Pacific coast of Canada and the United States. *Toronto, University of Toronto Press.* 207 pp. (1937).
9. Brinckmann-Voss, A. Seasonality of hydroids (Hydrozoa, Cnidaria) from an intertidal pool and adjacent subtidal habitats at Race Rocks, off Vancouver Island, Canada. *In*: S. Pirainno, F. Boero, J. Bouillon, P.F.S. Cornelius. Advances in hydrozoan biology. *Sci. Mar.* **60 (1)**, 89-97 (1996).
10. Carlton, J. T. (ed.) The light and smith manual: intertidal invertebrates from Central California to Oregon. *4th edn. University of California Press, Berkeley, CA*. (2007).
11. Migotto, A. E., Marques, A. C., Morandini, A. C. & Silveira, F. L. Checklist of the Cnidaria Medusozoa of Brazil. *Biota Neotrop.* **2(1)**, bn01102012002 (2002).
12. Oliveira, O. M. P., Miranda, T. P., Araujo, E. M., Ayón, P., Cedeño-Posso, C. M., Cepeda-Mercado, A. A., Córdova, P., Cunha, A. F., Genzano, G. N., Haddad, M. A., Mianzan, H. W., Migotto, A. E., Miranda, L. S., Morandini, A. C., Nagata, R. M., Nascimento, K. B., Nogueira Jr., M., Palma, S., Quiñones, J., Rodriguez, C. S., Scarabino, F., Schiariti, A., Stampar, S. N., Tronolone, V. B. & Marques. A. C. Census of Cnidaria (Medusozoa) and Ctenophora from South American marine waters. *Zootaxa* **4194**, 1-256 (2016).
13. Calder, D. R. Shallow-water hydroids of Bermuda: (Superfamily Plumularioidea). *R. Ont. Mus. Life Sci. Contrib.* **161**, 1-85 (1997).

14. Bogle, M. A. A review and preliminary revision of the Aglaopheniinae (Hydroida: Plumulariidae) of the tropical western Atlantic. *Master's thesis, 1984 University of Miami, Coral Gables*. 307 pp (1975).
15. Moura, C. J., Harris D J, Cunha M R & Rogers A. D. DNA barcoding reveals cryptic diversity in marine hydroids (Cnidaria, Hydrozoa) from coastal and deep-sea environments. *Zool. Scr.* **37**, 93–108 (2008).
16. Ansín Agís, J., Ramil, F. & Vervoort, W. Atlantic Leptolida (Hydrozoa, Cnidaria) of the families Aglaopheniidae, Halopterididae, Kirchenpaueriidae and Plumulariidae collected during the CANCAP and Mauritania-II expeditions of the National Museum of Natural History, Leiden, the Netherlands. *Zool. Verh. Leiden* **333**, 1–268 (2001).
17. Galea, H. R. New additions to the shallow-water hydroids (Cnidaria: Hydrozoa) of the French Lesser Antilles: Martinique. *Zootaxa* **3686 (1)**, 1–50 (2013).
18. Fraser, C. M. Hydroids of the 1932, 1933, 1935, and 1938 Allan Hancock Pacific Expeditions. *Allan Hancock Pacific Expeditions* **4**, 129-153 (1938).
19. Billard, A. Note sur l'*Antennularia antennina* Lin. et sur l'*A. perrieri* n. sp.. *Bull. Mus. Hist. Nat. Paris* **7**, 68-75 (1901).
20. Millard, N. & Bouillon, J. A collection of hydroids from Mozambique, East Africa. *Ann. S. Afr. Mus.* **65 (1)**, 1-40 (1974).
21. Boero, F. Systematics and ecology of the hydroid population of two *Posidonia oceanica* meadows. *Pubhlicazioni delict Stations Zoologica di Napoli I, Mar. Ecol.* **2 (3)**, 181-197 (1981).
22. Postaire, B., Magalon, H., Bourmaud, C. A. F., Gravier-Bonnet, N. & Bruggemann, J. H. Phylogenetic relationships within Aglaopheniidae (Cnidaria, Hydrozoa) reveal unexpected generic diversity. *Zool. Script.* **45**, 103–114 (2015).
23. Moura, C. J., Cunha, M. R., Porteiro, F. M. & Rogers, A. D. A molecular phylogenetic appraisal of the systematics of the Aglaopheniidae (Cnidaria: Hydrozoa, Leptothecata) from the north-east Atlantic and west Mediterranean. *Zool. J. Linn. Soc.-Lond.* **164**, 717–727 (2012a).
24. Svoboda, A. & Cornelius, P.F.S. The European and Mediterranean species of *Aglaophenia* (Cnidaria: Hydrozoa). *Zool. Verh. Leiden* **274**, 1–72 (1991).
25. Allman, G. J. Report on the Hydroida dredged by H.M.S. Challenger during the years 1873-76. Part 1.Plumularidae. Report on the Scientific Results of the Voyage of H.M.S. Challenger during the Years 1873-76. *Zoology* **7(20)** 1-54 (1983).
26. Cornelius, P.F.S. Medusa loss in leptolid Hydrozoa (Cnidaria), hydroid rafting, and abbreviated life-cycles among their remote-island faunae: an interim review. *Sci. Mar.* **56**, 245–261 (1992).
27. Cornelius, P. F. S. North-west European thecate hydroids and their medusae (Cnidaria, Leptolida, Leptothecatae). Part 2. *Synopses of the British Fauna, n.s.* **50**. 396 pp. (1995).
28. Galea, H. & Di Camillo, C. G. Rediscovery and redescription of *Gymnangium sibogae* (Billard, 1913) (Cnidaria: Hydrozoa: Aglaopheniidae). *Mar. Biodiv.* **47(3)**, 847-857 (2016).

29. Sars, G.O. Bidrag til Kundskaben om Norges Hvdroider. *Forh. VidenskSelsk. Kristiania* **1873**, 91-150 (1874).
30. Schuchert, P. Review of the family Halopterididae (Hydrozoa, Cnidaria). *Zool. Verh. Leiden* **309**, 1–162 (1997).
31. Dayrat, B. Towards integrative taxonomy. *Biol. J. Linn. Soc.* **85**, 407–415 (2005).
32. Heller, C. Die Zoophyten und Echinodermen des Adriatischen Meeres. *Verh. Zoologisch Botanischen Ges. Wien* **18**, 188 (1868).
33. Schuchert, P. Review of the family Halopterididae (Hydrozoa, Cnidaria). *Zool. Verh. Leiden* **309**, 1–162 (1997).
34. Nutting, C. C. American hydroids. Part I. The Plumularidae. *Spec. Bull. U. S. Nat. Mus.* **4 (1)**, 1-285 (1900).
35. Calder, D. R. Some shallow-water hydroids (Cnidaria: Hydrozoa) from the central east coast of Florida, USA. *Zootaxa* **3648 (1)**, 1–72 (2013).
36. Rees, W. J. & Thursfield , S. The hydroid collections of James Ritchie. *P. R. Acad. Edinb.* **69,** 34-220 (1965).
37. Schuchert, P. High genetic diversity in the hydroid *Plumularia setacea*: a multitude of cryptic species or extensive population subdivision? *Mol. Phylogenet. Evol.* **76**, 1–9 (2014).
38. Johnston, G. A history of the British zoophytes. *2nd ed. London, John van Voorst.* 488 pp. (1847).

Supplementary Tables

Supplementary Table S1. Sequence data and species delimitation analyses. (see Excel file in "Supplementary Information" section)

Supplementary Table S2. Number of sequences and nucleotide positions of each alignment used for the phylogenetic analyses.

	**Align. w/ gaps**		**Align. w/ some gaps**		**Align. without gaps**	
	**# Taxa**	**# Sites**	**# Taxa**	**# Sites**	**# Taxa**	**# Sites**
16S Dataset:						
**All Sequences**	1118	578	1118	376	1118	246
**Aglaopheniidae**	537	552	537	426	537	357
**Plumulariidae**	299	490	299	434	299	414
**Halopteridiidae**	218	553	218	444	218	308
**Kirchenpaueriidae**	59	493	59	458	59	427
16S+COI Dataset:						
**All Sequences**	251	808	251	642	251	614
**Aglaopheniidae**	122	859	122	699	122	584
**Plumulariidae**	89	807	89	779	89	722
**Halopteridiidae**	34	855	34	767	34	697
**Kirchenpaueriidae**	8	819	8	785	8	778

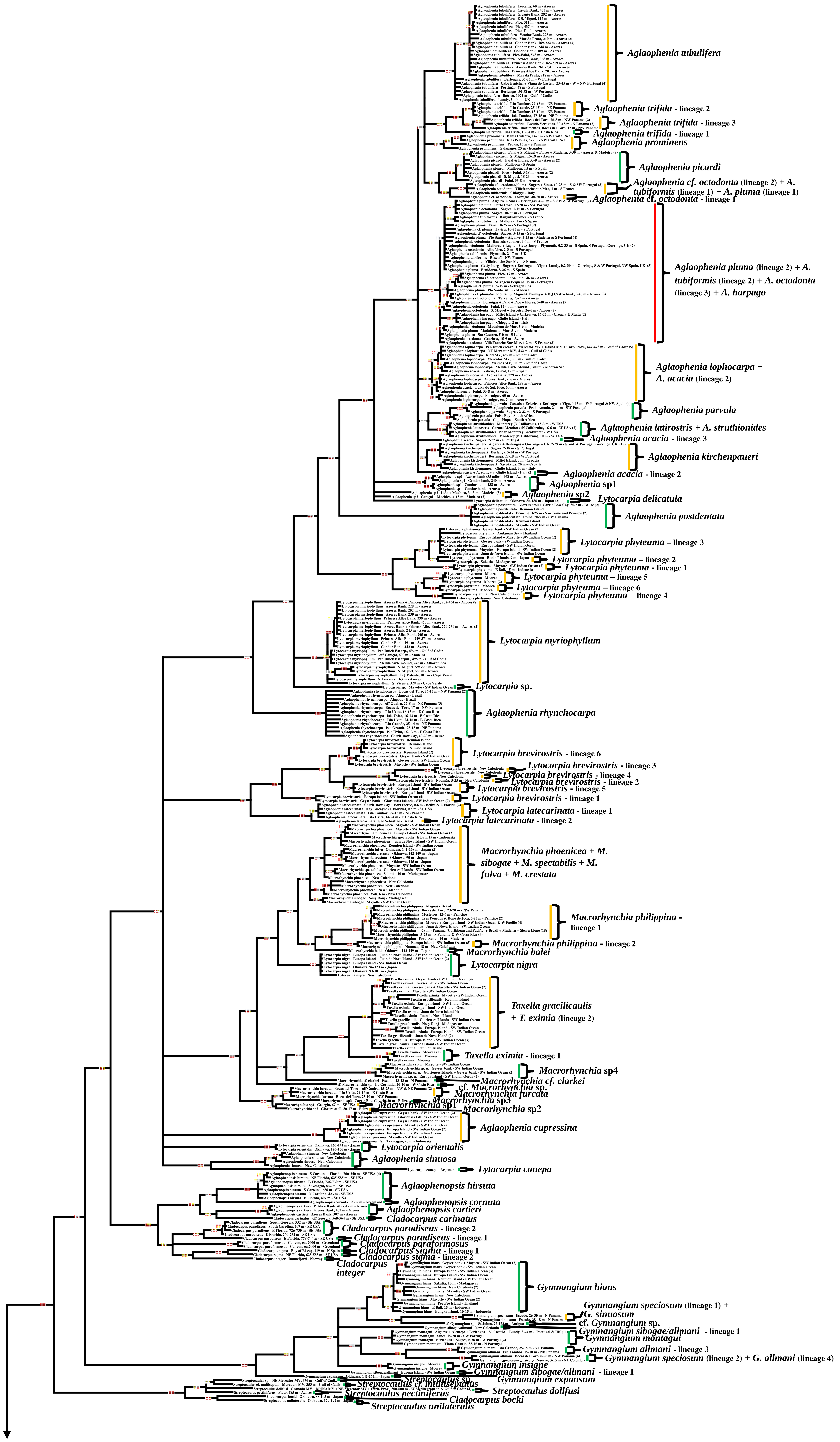
Aglaophenia tubulifera
Aglaophenia trifida - lineage 2
Aglaophenia trifida - lineage 3
Aglaophenia trifida - lineage 1
Aglaophenia prominens
Aglaophenia picardi
Aglaophenia cf. octodonta (lineage 2) + A. tubiformis (lineage 1) + A. pluma (lineage 1)
Aglaophenia cf. octodonta - lineage 1
Aglaophenia pluma (lineage 2) + A. tubiformis (lineage 2) + A. octodonta (lineage 3) + A. harpago
Aglaophenia lophocarpa + A. acacia (lineage 2)
Aglaophenia parvula
Aglaophenia latirostris + A. struthionides
Aglaophenia acacia - lineage 3
Aglaophenia kirchenpaueri
Aglaophenia acacia - lineage 2
Aglaophenia sp1
Aglaophenia sp2
Lytocarpia delicatula
Aglaophenia postdentata
Lytocarpia phyteuma – lineage 3
Lytocarpia phyteuma – lineage 2
Lytocarpia phyteuma - lineage 1
Lytocarpia phyteuma – lineage 5
Lytocarpia phyteuma – lineage 6
Lytocarpia phyteuma – lineage 4
Lytocarpia myriophyllum
Lytocarpia sp.
Aglaophenia rhynchocarpa
Lytocarpia brevirostris - lineage 6
Lytocarpia brevirostris - lineage 3
Lytocarpia brevirostris - lineage 4
Lytocarpia brevirostris - lineage 2
Lytocarpia brevirostris - lineage 5
Lytocarpia brevirostris - lineage 1
Lytocarpia latecarinata - lineage 1
Lytocarpia latecarinata - lineage 2
Macrorhynchia phoenicea + M. sibogae + M. spectabilis + M. fulva + M. crestata
Macrorhynchia philippina - lineage 1
Macrorhynchia philippina - lineage 2
Macrorhynchia balei
Lytocarpia nigra
Taxella gracilicaulis + T. eximia (lineage 2)
Taxella eximia - lineage 1
Macrorhynchia sp4
Macrorhynchia cf. clarkei
cf. Macrorhynchia sp.
Macrorhynchia furcata
Macrorhynchia sp3
Macrorhynchia sp1
Macrorhynchia sp2
Aglaophenia cupressina
Lytocarpia orientalis
Aglaophenia sinuosa
Lytocarpia canepa
Aglaophenopsis hirsuta
Aglaophenopsis cornuta
Aglaophenopsis cartieri
Cladocarpus carinatus
Cladocarpus paradiseus - lineage 2
Cladocarpus paradiseus - lineage 1
Cladocarpus paraformosus
Cladocarpus sigma - lineage 1
Cladocarpus sigma - lineage 2
Cladocarpus integer
Gymnangium hians
Gymnangium speciosum (lineage 1) + G. sinuosum
cf. Gymnangium sp.
Gymnangium sibogae/allmani - lineage 1
Gymnangium montagui
Gymnangium allmani - lineage 3
Gymnangium speciosum (lineage 2) + G. allmani (lineage 4)
Gymnangium insigne
Gymnangium sibogae/allmani - lineage 1
Gymnangium expansum
Streptocaulus sp.
Streptocaulus cf. multiseptatus
Streptocaulus dollfusi
Streptocaulus pectiniferus
Cladocarpus bocki
Streptocaulus unilateralis

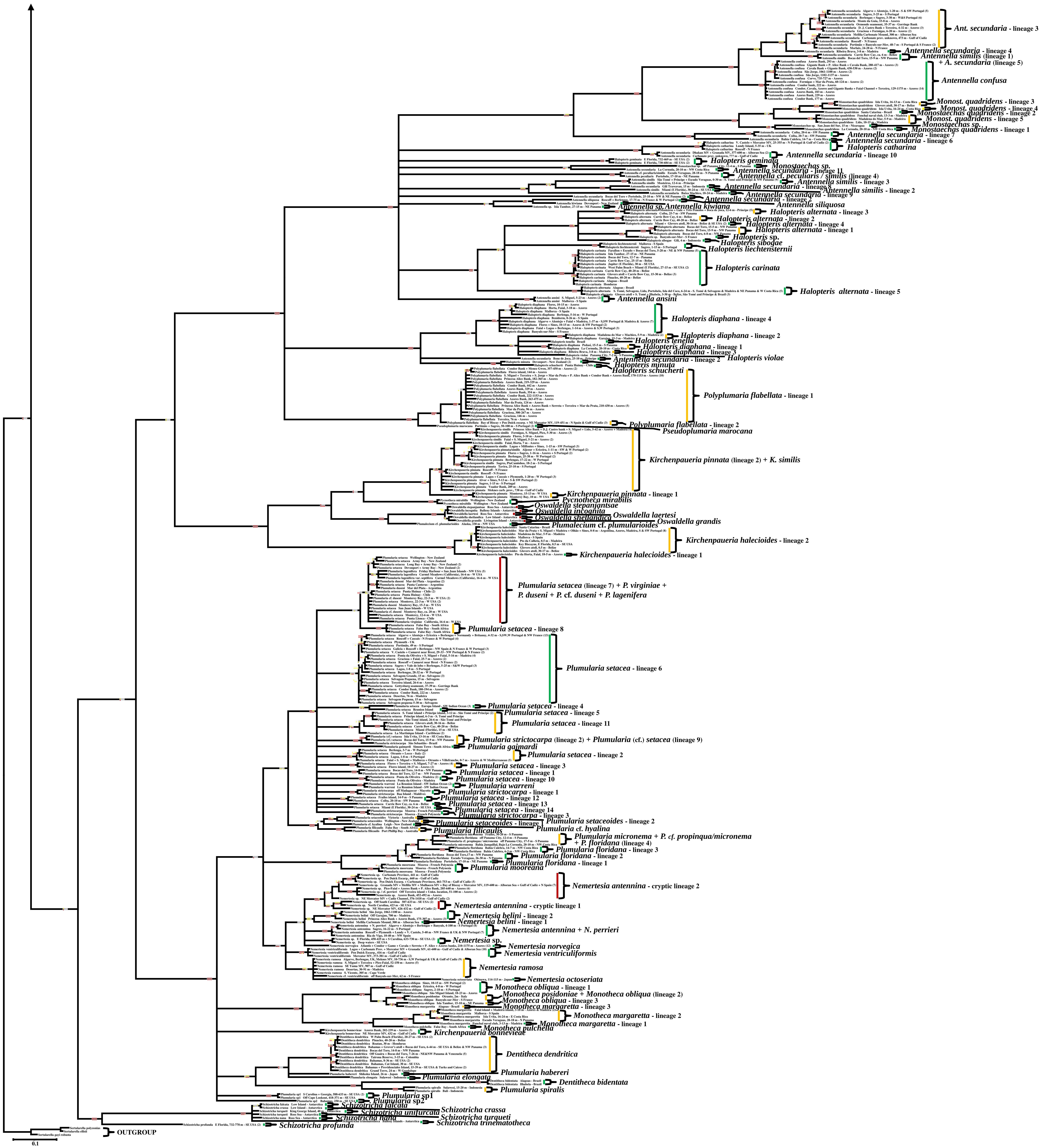

Supplementary Figure S1. Molecular phylogeny of hydroids of the superfamily Plumularioidea based on 16S sequence data, without Gblocks manipulations, obtained with MrBayes. Nodes collapsed presented posterior probabilities below 70% for the phylogenetic analysis represented graphically. Rectangles on nodes show nodal support (for ML analyses: yellow – 50-74%; red – 75-94%; dark-red – 95-100%; for Bayesian analyses: yellow – 75-89%; red – 90-97%; dark-red – 98-100%) for the different phylogenetic analyses: first horizontal row – ML; second horizontal row – Bayesian; first vertical row – alignment with all families together with gaps; second vertical row – alignment without manipulations per family; third vertical row – alignment with some gaps removed, per family; fourth vertical row – alignment without gaps, per family. The output of the species delimitation analyses (see also Supplementary Table S1) is resumed with the brackets; the colored bars associated represent the degree of confidence of these analyses (green - high confidence; yellow – medium confidence; red – low confidence).

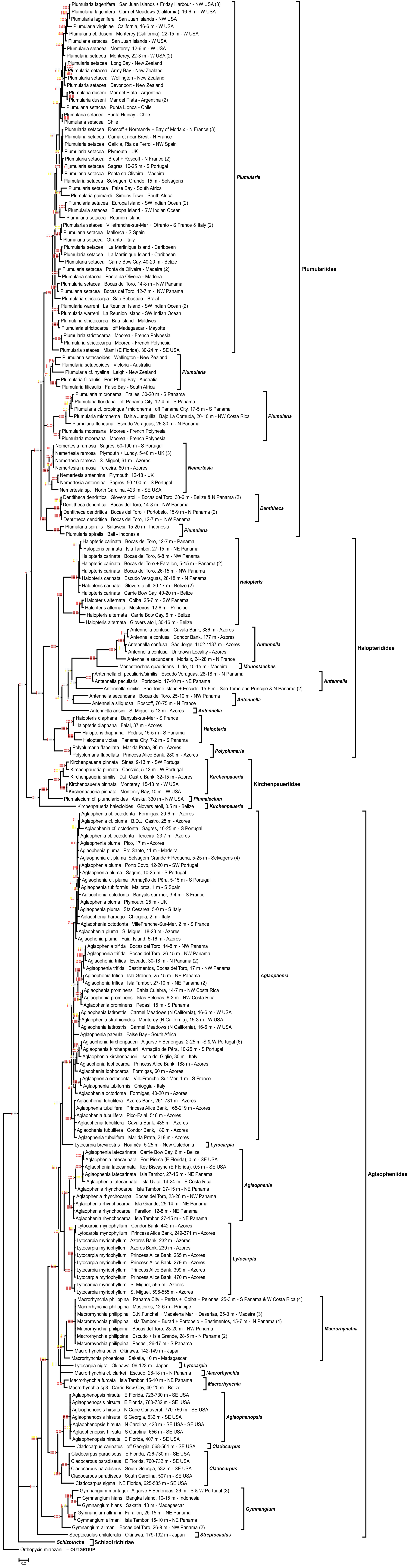

Supplementary Figure S2. Phylogeny of Plumularioidea hydroids based on 16S+COI sequence data, without Gblocks manipulations, obtained with MrBayes. Nodes collapsed presented posterior probabilities below 70% for the phylogenetic analysis represented graphically. Rectangles on nodes show nodal support (for ML analyses: yellow – 50-74%; red – 75-94%; dark-red – 95-100%; for Bayesian analyses: yellow – 75-89%; red – 90-97%; dark-red – 98-100%) for the different phylogenetic analyses: first horizontal row – ML; second horizontal row – Bayesian; first vertical row – alignment with all families together with gaps; second vertical row – alignment without manipulations per family; third vertical row – alignment with some gaps removed, per family; fourth vertical row – alignment without gaps, per family.
